# *In vivo* large-scale analysis of Drosophila neuronal calcium traces by automated tracking of single somata

**DOI:** 10.1038/s41598-020-64060-x

**Published:** 2020-04-28

**Authors:** Felipe Delestro, Lisa Scheunemann, Mélanie Pedrazzani, Paul Tchenio, Thomas Preat, Auguste Genovesio

**Affiliations:** 1 Computational Bioimaging and Bioinformatics, IBENS, ENS, INSERM, CNRS, PSL, 46 rue d’Ulm, 75005 Paris, France; 2 Genes and Dynamics of Memory Systems, Brain Plasticity Unit, CNRS, ESPCI Paris, PSL, 10 Rue Vauquelin, 75005 Paris, France

**Keywords:** Image processing, Software, Long-term memory

## Abstract

How does the concerted activity of neuronal populations shape behavior? Impediments to address this question are primarily due to critical experimental barriers. An integrated perspective on large scale neural information processing requires an *in vivo* approach that can combine the advantages of exhaustively observing all neurons dedicated to a given type of stimulus, and simultaneously achieve a resolution that is precise enough to capture individual neuron activity. Current experimental data from *in vivo* observations are either restricted to a small fraction of the total number of neurons, or are based on larger brain volumes but at a low spatial and temporal resolution. Consequently, fundamental questions as to how sensory information is represented on a population scale remain unanswered. In *Drosophila melanogaster*, the mushroom body (MB) represents an excellent model to analyze sensory coding and memory plasticity. In this work, we present an experimental setup coupled with a dedicated computational method that provides *in vivo* measurements of the activity of hundreds of densely packed somata uniformly spread in the MB. We exploit spinning-disk confocal 3D imaging over time of the whole MB cell body layer *in vivo* while it is exposed to olfactory stimulation. Importantly, to derive individual signal from densely packed somata, we have developed a fully automated image analysis procedure that takes advantage of the specificities of our data. After anisotropy correction, our approach operates a dedicated spot detection and registration over the entire time sequence to transform trajectories to identifiable clusters. This enabled us to discard spurious detections and reconstruct missing ones in a robust way. We demonstrate that this approach outperformed existing methods in this specific context and made possible high-throughput analysis of approximately 500 single somata uniformly spread over the MB in various conditions. Applying this approach, we find that learned experiences change the population code of odor representations in the MB. After long-term memory (LTM) formation, we quantified an increase in responsive somata count and a stable single neuron signal. We predict that this method, which should further enable studying the population pattern of neuronal activity, has the potential to uncover fine details of sensory processing and memory plasticity.

## Introduction

All behavior relies on accurate information processing of sensory signals carried out by the concerted activity of populations of neurons^1,2^. While much is known about the pathways of neuronal transduction, how the integration of information derives from the cooperative action of neuronal activity remains largely elusive. However, for many functions of the brain such as sensory processing or memory plasticity, the important coding might be represented within the neuronal population rather than the individual cell. In animal models, recent developments in the field of *in vivo* imaging tools have allowed neuroscientists to characterize neuronal activity patterns with unprecedented precision^3,4^. Yet the functional representation of sensory information in higher-order brain structures generally takes place in a subset of sparsely distributed neurons amongst a larger assembly and the main bottleneck comes from the lack of methodology to study task-specific biochemical processes in heterogeneous neuron assemblies.

Genetic approaches, including immediate early gene-based strategies to tag cells in an activity-dependent manner, have clearly advanced our understanding how neural activity underlies behaviour^5^. However, many of the current methods require fixed tissue to visualize previously active neurons and are therefore inadequate to follow fast temporal dynamics of activity patterns. Traditional *in vivo* metabolic imaging techniques like PET or fMRI to track neuronal activity with better temporal resolution lack the spatial resolution to address activity changes on a single cell level^3^. To achieve this, high spatio and temporal resolution calcium imaging using genetically-encoded fluorescent probes represents a powerful approach. Recent achievements to implement two-photon imaging i.e. in mouse or zebrafish made it possible to record the activity of hundreds - or even thousands - of neurons individually^6,7^. However, addressing structural changes with single cell resolution that occur within a whole neuronal assembly is difficult e.g. in rodent models because of the size of the brain structures like sensory cortices or memory-relevant structures like the hippocampus or neocortex^7,8^.

**Figure 1:**
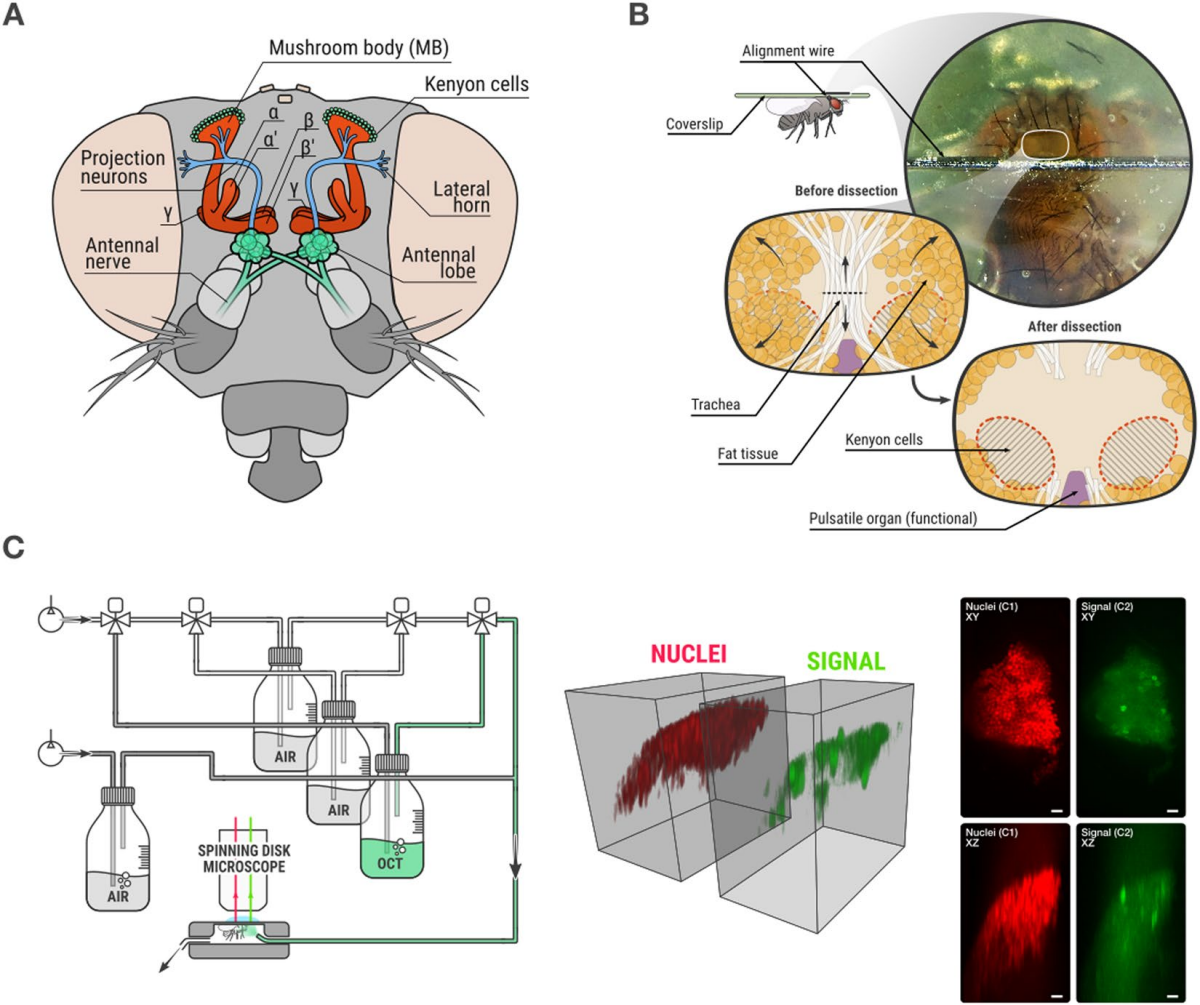
Biological model and data acquisition. (**A**) Frontal view of the *Drosophila melanogaster* olfactory system. Olfactory receptor neurons of the antennal nerve project to individual glomeruli of the antennal lobes, where they synapse with projection neurons and with local interneurons. From the antennal lobes, the olfactory information is conveyed by projection neurons to the mushroom body (MB), the olfactory learning and memory center, and to the lateral horn. Upon odor stimulation, a stereotypic pattern of glomeruli activity is observed in the antennal lobe, while the pattern of responses of Kenyon cells is not stereotypic because projections neurons are randomly connected to Kenyon cells in each fly. (**B**) Details of the dissection process, in which the MB is exposed for imaging. (**C**) Left: odor delivery system which simultaneously allows image acquisition and odor delivery to the fly. Middle: 3D volume rendering of a 3D stack after acquisition. Right: maximum intensity projections of nuclei and signal channels along Z axis (top) and Y axis (bottom) of a 3D stack. Scale bar is 10 µm.

The Drosophila model, which is compelling in respect of size, behavioral complexity and tractability of cellular processes, represents an ideal candidate to analyze the population code underlying sensory processing or memory formation^9–11^ (Fig. 1A). The long history in neuroscience from various disciplines gathered a level of detail in the understanding of the circuits and molecular pathways underlying information processing that is unmatched in the field^12,13^. The achievement of a full connectome profile at the single cell level of the Drosophila brain makes it possible to ask precise questions about the circuits that code and store information^14^.

The MB, a center of sensory processing and memory formation in the fruit fly *Drosophila melanogaster*, is small but highly organized^15^. Each MB hemisphere consists of approximately 2,000 Kenyon cells (KCs), whose cell bodies are densely packed at the MB calyx and whose axons first bundle within the peduncle and then form 5 discrete lobular structures (from the α/β, α’/β’ and γ neurons)^16^. The KCs receive input from cholinergic projection neurons that transmit odor information coming initially from olfactory receptors neuron in the antennae and the maxillary palps, which then relay their information to projection neurons in the antennal lobe^17^ (Fig. 1A). The ease of genetic manipulation in Drosophila has contributed to its development as a successful model organism, especially in the field of neuroimaging. Using *in vivo* imaging techniques with genetically encoded activity reporters, *i.e*. the calcium probe GCaMP^18^, odor responses have been demonstrated at the level of the KC cell bodies that can be detected with single-cell resolution^19^. The MB displays important features to model sensory and behavioral processes: its sparse and non-hardwired coding is thought to be essential for accurate sensory integration and learning, since it minimizes the overlap between input stimuli and allows for plastic changes^20^. Notably, the MB displays functional homologies to the hippocampus^21^, and its consolidation processes with respect to memory formation are very similar to those of humans and mammals^22,23^.

But despite the immense improvements of *in vivo* imaging techniques, recording neuronal activity of densely packed somata in Drosophila at high temporal and spatial resolution to achieve large-scale population analysis remains to date unreached. To overcome these limitations, we acquired 3D stacks of the Drosophila mushroom body (MB) at high speed using a spinning disk microscope^24^, developed a dedicated 3D tracking algorithm to detect single cells from a neuronal network and monitored activity changes from the cell body of individual neurons. Thus, to demonstrate the power of this novel approach, we questioned if memory plasticity could be observed on a population scale by MB cell body activity patterns.

## Results

### 3D+ time confocal imaging of mushroom body somata *in vivo*

The Drosophila olfactory system represents a well-established model to analyze sensory processing, especially how information is integrated into higher-order structures like the MB (Fig. 1A)^17,25^. Here, in order to image a large cellular population with a spatial and temporal resolution that allows detection of activity from single cell bodies, we needed to overcome the speed limitations imposed by point scanning and two-photon microscopes. To accomplish this, we used a spinning disk microscope that offered a faster acquisition at the cost of a slightly lower resolution^24^. To get optical access to the MB cell body layer, we performed *in vivo* brain preparations. Therefore, flies were affixed ventral side up on a coverslip and the head position was stabilized using an alignment wire (Fig. 1B). We then turned the preparation over to open a small window in the coverslip, allowing us to open the cuticle at the back of the head while leaving the rest of the fly intact^18^. Underneath the cuticle, the brain is surrounded by fat tissue and a thin layer of trachea that hinder direct observation of the KC cell bodies. In order to maintain a high degree of normal brain function, we gently cut the trachea and pushed both the fat and trachea aside, without removing either tissue (Fig. 1B). Another important issue is the pulsatile organ (*i.e*. the heart tube), which terminates at the posterior edge of the brain. The rhythmic activity of the pulsatile organ can strongly move the brain, which is deleterious for *in vivo* brain imaging. Contrary to our procedure, imaging studies that aim single cell resolution disrupt the physiological function of the pulsatile organ by disconnecting its innervating muscles, which may affect brain physiology^19^. Interestingly, our dissection technique prevents strong activity in this organ, and the remaining fat tissue serves as a physiological buffer that minimizes shifting of the brain. Using this procedure, we obtained a level of brain movement that could be corrected using image processing, allowing us to record brain activity in the presence of a functional pulsatile organ (Fig. 1B).

This fly preparation provides direct optical access to the MB neurons. Taking advantage of the precise genetic targeting techniques available in Drosophila, we co-expressed the nucleus marker NLSmcherry (red) and the calcium sensor GCaMP6f (green) in all KCs using the VT30559-GAL4 driver, for subsequent cell detection and response analysis, respectively. This step ensures that only a small part of the brain is labeled, minimizing the out-of-focus background. We confirmed that expressing these fluorescent reporters in the MB did not interfere with normal behavioral performances by testing LTM formation (Supplementary Fig. 1). Next, the fly preparation was positioned within a custom-built odor delivery system under the microscope (Fig. 1C). This system uses serial air dilutions to maintain a constant airflow of 1.25 L/min at the level of the fly’s antennae. Switching between clean and odorized air streaming using synchronous two-way valves creates odor or air pulses; the odor concentration was thus set to 1/500. The open head capsule, covered in Drosophila Ringer’s solution and fully isolated from the odor delivery system, was positioned under the objective of the microscope (Fig. 1C). All flies were exposed to the same sequence of stimuli, alternating between 5 s of odor stimuli or air control pulses and 35 s of no stimuli (see online methods). The short stimulus response duration for odorants in the MB imposed a high frequency of 3D stack acquisition, thus defining the maximum number of 2D images that could be obtained in a 3D stack at a given time point. Conversely, in order to obtain the most information given the resolution limit imposed by the point spread function (PSF) in the axial direction, a maximum z-step size between 2D images was imposed (see online methods for details). After optimization, acquisition of the entire population of the Drosophila memory center (*i.e*. the KC somata of the MB) was made possible. We acquired both spectral channels in parallel, with red NLS mcherry labeling for KC nuclei and green GCaMP6f for calcium activity. By using an acquisition speed of 20 ms per 2D image, we obtained 3D stack of 45 2D images every 900 ms. This ensured that the 3D stack covered the whole 3D zone of the KC somata continuously, with z-step size of 1.5 µm between two 2D images. This also ensured that any event lasting longer than 900 ms could be captured. The volume rendering of a raw 3D stack as well as some maximum intensity projections are displayed in Fig. 1C and Supplementary Video 1. This demonstrates that imaging the entire Drosophila memory center, *i.e*. the MB cell body network, at a single-cell resolution is feasible, and opens the door to an unmatched analysis of full population activity dynamics of sensory representations in the MB.

### Fully automated monitoring of densely packed single somata activity

After image acquisition, a single 3D movie consisted of 120 consecutive 3D stacks of 45 2D images each. The tracking algorithm consisted of four steps: 1) anisotropy correction of 3D stacks to limit the impact of the low resolution in the axial direction, 2) spots detection, 3) rigid and non-rigid registrations of these detections over time to group them by somata, thus limiting the impact of spurious and missing detections, and 4) detection of these clusters in 3D + time to reconstruct and complete individual trajectories.

**Figure 2:**
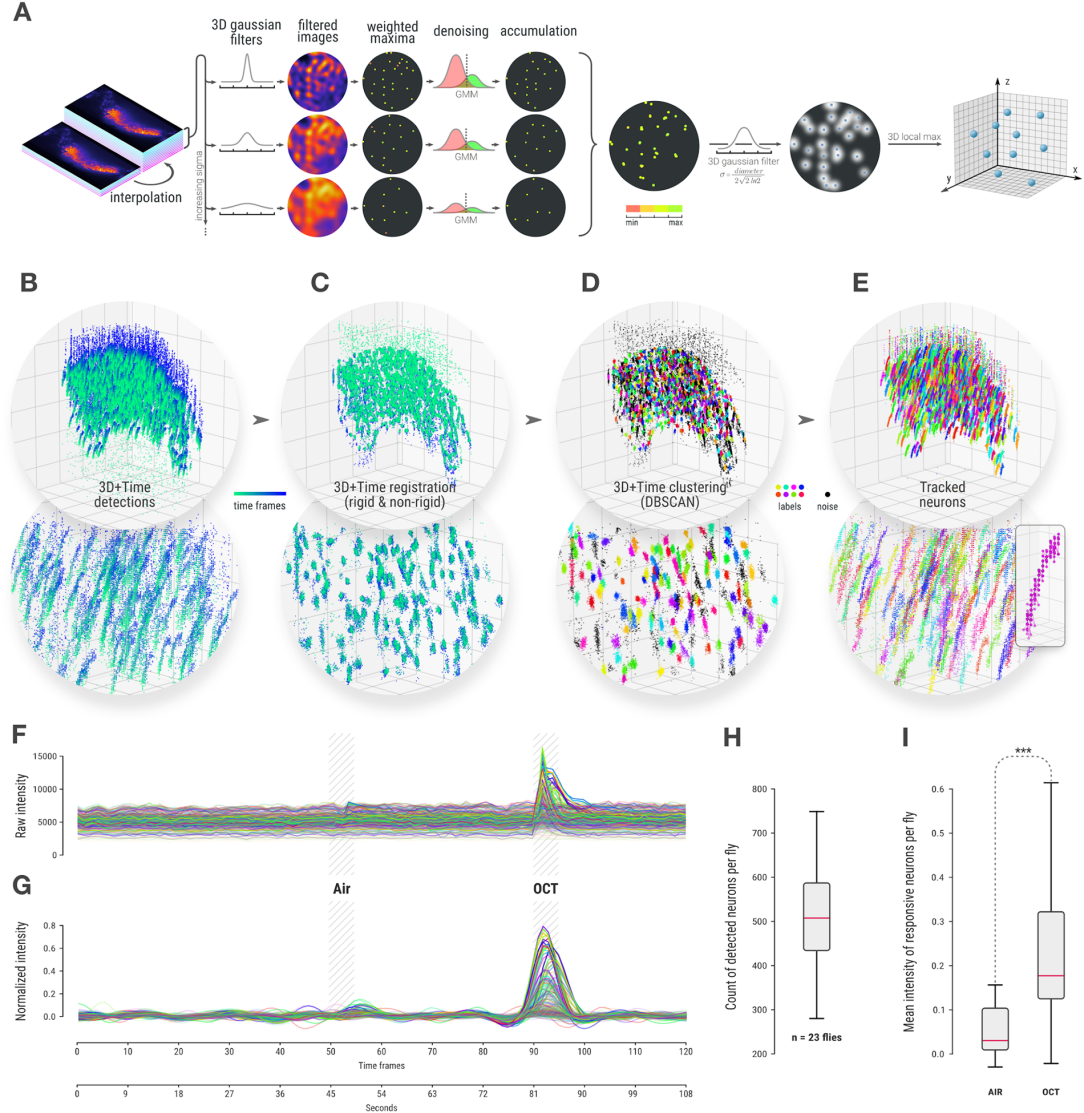
Detection, tracking and signal measurement for densely packed single-neuron analysis. (**A**) 3D nuclei detection method with anisotropy correction, multiscale detection and filtering that produces 3D spots locations. While we show that results are more accurate than the one obtained with available spot detection methods for this task, they still contain spurious detection or regularly miss detecting nuclei along the whole sequence leading tracking software to fail in this context. (**B**) Detected positions of nuclei in 3D for every time frame of a 3D + time sequence grouped together. Colormap represents time and the bottom image is a zoom in the center of the cloud. (**C**) The same 3D + time point cloud after rigid and non-rigid registrations forms visible clusters. (**D**) Clustering of dense region by DBSCAN enables to identify those clusters and remove noise. (**E**) Final nuclei trajectories in the original spatial coordinates are identified by successive individual detections over time with the same cluster id (here the same color). Merged trajectories and missing detection can easily be reconstructed at this point (see Supplementary Fig. 4) (**F**). For each detected nucleus, the raw GCaMP signal is measured over time in a volume around the nuclei. 500 individual neurons signal from the same MB cell bodies are displayed here. (**G**) Normalized signal displayed only for responsive neurons selected as having a peak above 0.1 (see online methods). (**H**) Count of responsive neurons per fly for a group of 23 naïve flies. (**I**) Mean intensity of responsive neurons per fly during air and OCT stimulation windows showing a highly significant difference (Wilcoxon signed-rank test, p-value: 7.3089e-68).

The first step consisted in correcting the anisotropy of 3D stacks. The uneven shape of the 3D Point Spread Function (PSF) and the distance between two 2D images (1.5 µm) being 10 times larger than between two pixels within a 2D image (0.16 µm), resulted in the resolution being much lower in the z axis than in the x/y axes. Anisotropy of 3D stacks in fluorescence microscopy is common. However, it is often overlooked in subsequent image analysis leading to suboptimal precision of 3D spot detection algorithms, especially in the axial direction. The first step of our approach lowers down the aforementioned effect: void between consecutive 2D images in a 3D stack was filled with 9 interpolated 2D images using cubic splines (see the left panel of Fig. 2A)^26^. This could not correct for the anisotropy of the PSF but produced cubic voxels more relevant to the isotropic 3D convolutions performed in the subsequent spot detection.

The second step consisted in spot detection. In order to capture small variations in nucleus volume, the interpolated 3D stacks were convolved with a bank of 10 isotropic 3D Gaussian filters ranging in size around the average nucleus diameter. This process produced, for each input 3D stack, 10 output 3D stacks (one per filter), each emphasizing a slightly different spot size (see Fig. 2A). The average nucleus size, the sole input parameter of this step, could easily be estimated from isolated nuclei by measuring the intensity profile and calculating the Full Width at Half Maximum (FWHM). For the actual spot detection, 3D local maxima were then identified from each of those output 3D stacks, based on a neighborhood of 3×3×3 voxels. The identified spots are then partitioned into background noise or actual nuclei signal thanks to a two-component Gaussian mixture model (GMM) fit based on their intensity level. Indeed, local maxima in theory should follow a bimodal intensity distribution since those located on nuclei are expected to be significantly brighter than those found in the background noise. This automated thresholding process thus separates the foreground and background detections. Remaining maxima, corresponding to nuclei on each filtered 3D stack, were drawn with their original intensity into a single empty 3D stack. This aggregated signal was then merged by convolution with a Gaussian filter matching the average nucleus size. These processes, at the end, enabled the reconstruction of a 3D stack with denoised spots. 3D maxima locations (x,y,z) extracted from these artificial 3D stacks were found to be much closer to the ground truth than the one obtained with any other existing methods, as assessed by comparison made on synthetic simulations and on manually annotated data (see Fig. 2A describing the spot detection method and Supplementary Fig. 2 describing the evaluation against other methods).

Following this step, we seek to link spot detections to reconstruct individual somata trajectories. Unfortunately, we found that available multi-target tracking software failed to properly resolve trajectories in this context. We observed that those poor performances were mainly due to the inability of broad spectrum tracking methods to reconstruct a correct linkage in the presence of a large amount of missing and spurious detections. In order to overcome these issues and obtain reliable trajectories from this large set of scattered detections, we took advantage of a hypothesis met by our data: the MB cell body layer being a tissue, even if the fly can move as a whole with erratic movements and the tissue can bend locally, the distance between any two close by somata remains almost constant during the sequence acquisition. For instance, we do not expect somata to move in a way that they could cross each other or exchange positions. Registering 3D point sets first as a whole could then help recover missing detection or identifying merged trajectories. Therefore, we performed the tracking in two steps: a registration of the whole 3D + time sequence followed by a clustering of this registered point set to resolve identity of detections and thus correct for missing and spurious detections in the way. The two steps, registration and clustering, are described in the two next paragraphs and further details are given in the method section.

As a third step, a rigid registration between all detected 3D points at all time steps was first performed using chained affine transformations of all 3D stacks of a sequence together to remove the global shift due to the large movements of the fly (Fig. 2A,B and online methods). Then, this coarse alignment was followed by a more precise non rigid registration using the Coherent Point Drift algorithm^27^ (Fig. 2C and online methods). After this procedure, all detections over time for every single somata resulted in a more clearly identifiable cluster, even if they would not contain detection for all time steps. Each of these clusters in this 3D + time registered dataset corresponded to a single trajectory in the unregistered images.

In a fourth and last step, the actual identification of each cluster was subsequently obtained by using DBSCAN on the 3D + time registered dataset (Fig. 2D)^28^. DBSCAN is a clustering method that offers several advantages in our particular case. It automatically identifies the number of clusters, takes into account the local density and disregards isolated points considered as spurious detection or background noise without associating them to a group. Optimal DBSCAN parameters were estimated from the data (Supplementary Fig. 3). Once each detection was either associated with a cluster, and therefore with a trajectory, or dismissed as isolated background noise, trajectories of individual somata could be recovered in the original coordinates (see Fig. 2E). The clustering algorithm uses only the spatial information from the detected spots, ignoring their temporal position. This possibly resulted in clusters containing two or more spots at the same time frame, meaning two (or more) somata were merged in the same cluster. We use the temporal information to split these N merged objects by identifying those clusters containing about N times the expected amount of detections. Clusters were thus split and trajectories individually recovered (Supplementary Fig. 4). After this step, missing detections in each trajectory could be reconstructed by interpolation of close detections in time of the same somata (see reconstruction of trajectories and missing detections in the methods section).

A quantitative comparison of our tracking algorithm using both manually annotated and synthetic datasets demonstrated that our approach outperforms currently available software for this task (see tracking evaluation in online methods and Supplementary Figs. 5–7).

Once the tracks of individual neurons were obtained, it was possible to measure their individual signal through time. Comprehensive extraction of the raw signal intensity in the GCaMP channel was obtained by gathering the average intensity in non-intersecting volumes around all successive positions along each trajectory. This step employed a discrete 3D Voronoi tesselation seeded by the tracked somata. Figure 2 shows the raw (Fig. 2F) and normalized (Fig. 2G) signals for all neurons tracked in a single fly (see online methods for signal normalization). Since the process was fully automated, it was possible to run the same analysis in parallel on many fly acquisitions using a computing cluster. As a validation step, 23 naïve flies that had never been exposed to octanol (OCT) were each exposed to a 5 s odor stimulus (OCT) or a control air puff stimulus as described in Fig. 2F–G (see online methods for full details). The full sequence was simultaneously imaged and automatically analyzed.

The results indicate that an average of 500 somata per fly were tracked (Fig. 2H). From our evaluation on manually annotated and synthetic datasets, we could assess that most of them were true positives matching real neurons (see Supplementary Fig. 6). Furthermore, comparing the cell body count responding in the air window versus the neuron count responding in the octanol window revealed a significant difference, demonstrating that the odor signal could be captured by monitoring the single-cell signal of KC soma (Fig. 2I).

Furthermore, we asked if the activity pattern of odor responding somata detected by our algorithm would allow us to differentiate between two different odors presented sequentially to the fly. Next to OCT, we chose methylcyclohexanol (MCH) as the second odor, since this pair of odors is used in our olfactory memory assay hereafter, as well as in several other studies on MB odor processing^13,14^. Odor processing is not hardwired in the MB, nevertheless, each odor is supposed to elicit a specific pattern of KCs in the individual naive fly. Therefore, we presented both odors OCT and MCH two times each to the same fly and tested if KC somata patterns were indeed significantly more similar between presentations of the same odor than between presentations of different odors. To that end, we calculated the Jaccard index of responding KC somata between two presentations of the same odor presentation or between presentations of different odors for each of 23 tested flies. The Jaccard index is a similarity measure between two sets represented by the ratio of the intersection of two sets divided by its union (Supplementary Fig. 8A). Indeed, the Jaccard index computed between presentations of the same odor was significantly higher than the Jaccard index computed between presentations of two different odors (Supplementary Fig. 8B). To avoid biases, we alternated the odor presentations starting randomly with OCT or MCH. The time trace sequence from one example fly shows the responses of individual KCs to OCT and MCH as well as to Air control in the beginning (Supplementary Fig. 8C). Clustering the responses using a cosine distance matrix clearly group presentations per odors on this example (Supplementary Fig. 8D). Together, the automated tracking algorithm developed in this study was able to classify the response pattern of different odors.

### Quantitative population analysis of odor responses after LTM formation

Using a well-established conditioning paradigm, Drosophila can robustly learn to avoid an odor that was previously paired with electric shocks^16^. Several distinct memory components have been identified that differ in their temporal dynamics as well as the underlying circuits and pathways^16,29^. Stable protein synthesis-dependent long-term memory (LTM) is exclusively formed when fruit flies are exposed to 5x spaced training cycles, which is a specific conditioning pattern during which the training cycle is repeatedly experienced with intervening resting intervals^30^. Several studies have found activity signatures that are specific to LTM in the MB^31,32^. In detail, these studies identified increased calcium responses to the trained odor, which is defining an LTM engram, in the axons of specific subpopulations of KCs, representing an early LTM-specific activity signature in α/β neurons and a late-phase signature in γ neurons. Nevertheless, these studies analyzed large sets of neurons at the MB lobe level, and measured a global increase in odor response intensity after LTM training. To challenge our 3D imaging and neuronal tracking technique, we asked the question if the LTM engram is represented on a population level, i.e. by a change in number or distribution of odor-activated cells after training. MB neurons at the axonal level are beyond individual resolution of current 3D *in vivo* imaging approaches^33^. Therefore, we focused on the cell body layer of the MB to identify activity patterns of individual somata. Since cellular calcium signals should reflect back-propagating neuronal activity from the axons, response patterns of MB cell bodies to a learned odor are thought to represent the plasticity changes that have occurred during memory formation.

**Figure 3:**
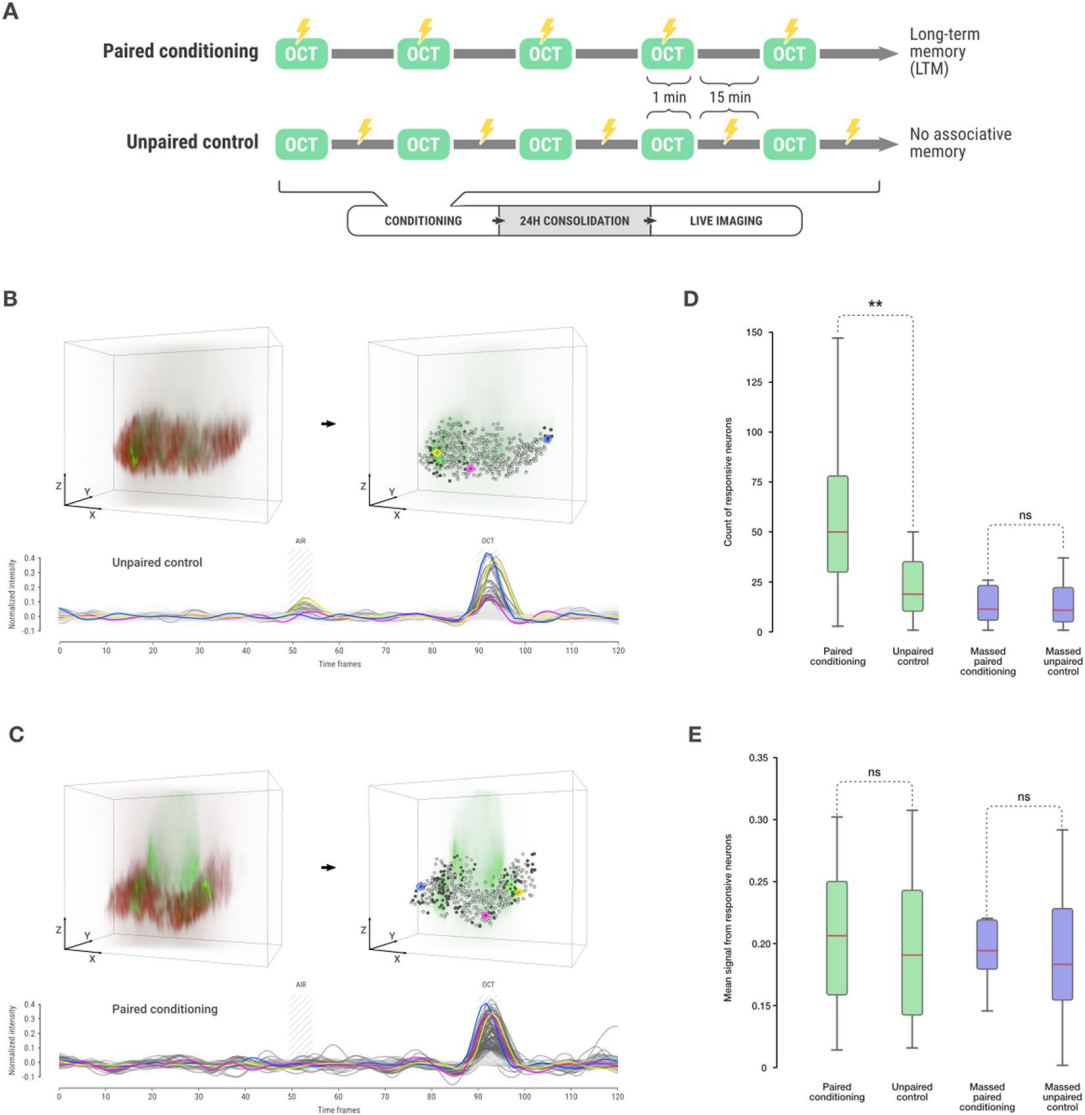
Detection of responsive neurons from spaced and massed trained flies. (**A**) LTM conditioning consists in a repeated presentation of an odor paired with electric shock. (**B**) An illustrative sample from the unpaired control group. The volumetric reconstruction on the left shows the mCherry nuclei signal together with GCaMP activity during the OCT response. On the right, the GCaMP signal is shown together with the detected nuclei. The plot shows the normalized signal from the individual neurons. Light gray spheres and lines represent neurons that did not respond, and the dark gray color indicates neurons that responded. Three neurons are highlighted in pink, yellow and blue (with the corresponding signal of the same color) to show that the signal of individual neurons can be monitored in different parts of the mushroom body. (**C**) An illustrative sample from the paired conditioning group using the same visualization as in panel B. (**D**) Comparison of the numbers of responsive neurons per fly between the paired 5x spaced conditioning to form LTM (n = 29) and the unpaired control (n = 27) groups (Mann-Whitney two-sided test p-value: 0.001098) as well as between the paired 5x massed conditioning to form LT-ARM (n = 14) and the unpaired control (n = 16) groups (Mann-Whitney two-sided test p-value: 0.97894). Results show that the number of responsive neurons increases after paired conditioning but not after massed paired conditioning. (**E**) Comparison of the mean intensity of responsive neurons per fly at stimulus time in the same flies and conditions as in panel D. Statistical tests could not reject the null hypothesis of an equal mean distribution of intensity (t-test p-value 5x spaced paired vs unpaired p-value: 0.882074 and t-test p-value 5x massed paired vs unpaired p-value: 0.5291736).

To induce LTM, flies were exposed to the 5x spaced training cycles of octanol and electric shock pairings that are classically used to induce LTM (Fig. 3A). As an unpaired control, a second group of flies received non-overlapping odor and shock presentations, which does not allow for the formation of associative memory (Fig. 3A). After the 24-h consolidation phase, flies were dissected and the entire KC cell body population was imaged as previously described for the naïve group. Here, presenting octanol during image acquisition resembles the situation during memory retrieval. Data analysis was performed automatically, identically and in parallel for the two groups.

A minority of flies displayed either no clear increase of neuronal activity in response to the odorant presentation, or non-specific KC activity. We therefore developed an automated quality control for odor responses that we used as a filter to identify responsive flies, and we excluded non-responsive flies from our analysis. The rate of validated flies was similar among the different conditions: out of 36 fly acquisitions in the paired condition and 40 in the unpaired condition, a total of 29 paired flies and 27 unpaired flies passed our automated quality control (Supplementary Fig. 9). The results from the automated analyses are presented for two examples from the unpaired control group (Fig. 3B) and the paired group (Fig. 3C). The volumetric reconstruction of the KC cell bodies shows the GCaMP activity during the octanol (OCT) response window, with time traces showing the normalized signal from the individual neurons over the whole acquisition time (Fig. 3B,C). At any time-point, the response level of an individual neuron can be assessed and located within the KC population (Fig. 3B,C, additional examples can be seen in Supplementary Fig. 10 and Supplementary Video 2). When comparing responsive somata from the paired and unpaired groups, more somata seem to respond to octanol in the paired group. Indeed, plotting the count for responsive cell bodies from both groups demonstrates that the number of cell bodies responding to octanol in the paired group is significantly higher (and almost doubled) as compared to the unpaired control (Fig. 3D). In addition, the mean intensity of the responses was not significantly different between the paired and unpaired groups (Fig. 3E).

We wanted to exclude the possibility that this effect could result from the fluorescent glow produced by an increased GCaMP signal in neighboring cells. If this were the case, an increased response intensity would create an artificial increase in the responsive cell body count. To test this hypothesis, we temporarily excluded the signal of several responses from the analysis, such that none of the remaining neurons could be closer to one another than a distance equivalent to twice the size of an individual soma. When iteratively removing somata that were too close to one another, the dimmest was always selected first in order to remain consistent with our hypothesis. Once the responses were filtered in this way, the cell body count for the octanol-paired group remained significantly higher than for the unpaired group (Supplementary Fig. 11). This demonstrates that the increase in cell body count cannot be explained by a glowing effect due to a hypothetical increase in the intensity of the GCaMP signal.

To control if this increased neuron count from the wild-type octanol-paired group could be specific to the trained odor and not due to the LTM conditioning in general, we trained flies to pair an electric shock with a second odor, methylcyclohexanol (MCH). Again, we trained one group by pairing MCH with shocks, and a second unpaired control group received MCH and electric shocks at separate time points. During image acquisition, OCT was presented to the flies 24 h later and the responsive cell count was not found to be significantly different (Supplementary Fig. 12). Thus, the increase in responsive MB somata observed in Fig. 3D was specific to an LTM association between OCT and electric shock.

A hallmark of LTM across phyla is the requirement of repeated training cycles with resting intervals, while the presentation without resting intervals, i.e. massed training, will not lead to protein-synthesis-dependent LTM^34^. In flies, 5x massed training leads to a memory called long-term anesthesia-resistant memory (LT-ARM) that is not sensitive to the inhibition of translation^30^. We therefore tested the neuron count of odorant-responsive cells after 5x massed training and again compared the paired with the unpaired group as it was done for 5x spaced training (Fig. 3D). This time, no increase in the number of responsive cell bodies was observed for the paired 5x massed group demonstrating the specificity of additional responding KC cell bodies to LTM formation (Fig. 3D). Importantly, in all of these tests, the number of KC detected using the mCherry reporter did not differ between the different conditions (Supplementary Fig. 13).

Altogether, we described a 3D imaging and automated response tracking method for a higher order neuronal ensemble that allows quantitative and qualitative analysis of population coding for sensory information. We demonstrate that we robustly capture odor responses that we can assign to individual and identified somata of the whole MB cell body layer. By using this system, we identified that more MB somata respond to the trained odor specifically after LTM formation, indicating that memory plasticity is reflected on a population scale in the MB.

## Discussion

In this work, we implemented 3D live imaging of the *Drosophila melanogaster* MB cell body population. The sparsity of the somata responses dictated that a 3D volume containing the whole MB cell bodies should be acquired at single-cell resolution, and at a time frequency fast enough to image the odor responses. These strong constraints pushed the spinning disk confocal imaging to its limits and produced 3D stacks containing many densely packed fluorescent spots. For this reason, available generic spot detection methods produced highly noisy and inaccurate results, which did not permit obtaining reliable 3D positions of somata^35–37^. As a consequence, none of the current state of the art algorithms used to track multiple spots through time could produce satisfactory results. To address these issues, we developed dedicated methods for densely packed spot detection and tracking and demonstrated quantitatively that it outperform other approaches. The reason why our approach performs significantly better than generic tracking method is that it benefits from our specific context. In this context, individual somata movement are not independent and move together as a neighborhood. Therefore, even if spot detection on a single frame produces noisy and incomplete result, registering the whole set of detection together overtime enables us to form clusters even in case of a large amount of missing detections and noise. The drawback is of course that this method applies to this context or could extend to tissue in general but is not adapted to track freely moving objects such as endosomes or viral particles.

Automatic calcium imaging image analysis algorithms were proposed for mammalian brains, but they are not adapted for the Drosophila brain. For instance, suite2p is well adapted to two photons images but this modality would not be fast enough to acquire the whole 3D MB at the required frequency to retrieve single somata signal in 3D^38^. Furthermore, a powerful method based on constrained deconvolution approach necessitates a much faster acquisition than achievable when imaging the whole MB^39^. Also, it was proven to work in 3D using light sheet microscopy on Zebrafish, but this modality would be impossible to employ in our context as the MB is not transparent. A central point in our tracking approach was to operate a 3D spot detection on all 3D stacks of a sequence, then employ a strategy to register these points set together in order to transform confused trajectories to identifiable clusters. As a rich literature exists on MRI image registration, we wondered if the opposite strategy, i.e. registering 3D stack first then detecting spots, could be an option^40,41^. It turned out to be inefficient as image transformation reduced further the signal content of 3D stacks and further decreased accuracy of spot detection. Similarly, retrieving signal intensity out of a transformed image was in principle less satisfying than retrieving signal intensity directly from the original images as we do. Furthermore, we aimed at not only registering two 3D stack together but a whole bunch of them to a reference frame in a sequence that included important spatial drifts and deformations which these approaches are not designed to deal with. Finally, detecting spots then registering detection is a lighter process making the overall method much faster.

Our approach made it possible to robustly recover the signal of approximately 500 single neurons from the whole MB cell body layer for each one of the 216 flies *in vivo*. This level of throughput, which has never been attained before in Drosophila, offers new perspectives as it is large-scale (encompassing the whole MB cell bodies) and can operate with individual cell body precision. Although the MB contains approximately 2,000 neurons per hemisphere, our imaging conditions could only successfully resolve about a 1,000 cell bodies by manually counting NLS-mCherry-positive neurons (Supplementary Fig. 2C: TP + FN in Smax represent the manual count of all nuclei in one 3D stack at a fixed time-point). Furthermore, synthetic simulations demonstrated that when processing artificial MB populated with 2,000 labeled neurons, our system was able to track robustly about a 1,000 neurons, almost all of them being true positive (see Supplementary Fig. 2D). Therefore, we estimate that our approach, limited by the resolution constraint, is capable of tracking correctly about half of the 1,000 manually identified MB somata within our acquisitions and do not generate false positives. In total, this suggests that we can locally detect about one in four cell bodies. Nevertheless, this subsampling is uniformly distributed over the whole mushroom body, and provides access to a broad view with unprecedented precision. Moreover, given that we were able to capture the GCaMP signal from the entire MB cell body layer, we are confident that our method can be used to describe a comprehensive response pattern of MB activity to a given odor.

The activity patterns obtained in this work suggest that LTM formation is supported by a recruitment of additional neurons that respond to the trained odor during retrieval. To strengthen this hypothesis, additional experiments will be needed; i.e. training under the microscope to directly compare activity dynamics of responding cell bodies before and after training in the same animal. Several recent studies aimed to identify LTM engram cells in the Drosophila MB *ex-vivo*, mainly by targeting neurons using biochemical processes specific for LTM^42–44^. The combination with our *in-vivo* imaging technique will enable us to reveal even more precise mechanisms of a memory consolidation in Drosophila. The questions about the ongoing reorganization and recruitment of neurons into the LTM engram is indeed highly debated^45,46^. In recent years, the combination of transgenics and optogenetics has allowed neuroscientists to identify memory engram cells by detecting and tagging specific populations of cells that are active during different learning phases^5^. In agreement with our results, one recent study found an activation of neurons in the prelimbic cortex during the retrieval of fear memory in mice in addition to those neurons active during stimulus presentation or early memory recalls^47^. However, this study, was performed in fixed samples, which lack temporal resolution and do not permit a qualitative analysis of the signals. In contrast to these findings, other studies have demonstrated that a consolidated memory trace, representing memory-dependent structural or molecular changes, can be restricted to neurons that were previously activated by the stimuli presented during associative learning^45,46^. The presented technique in this study could help reveal some of the general rules underlying the population code of LTM plasticity. Along these lines, one interesting direction for future studies would be to determine the dynamic evolution of the recruitment of MB neurons during memory consolidation.

Altogether, we used the Drosophila MB as a unique olfactory network model and developed an imaging and activity tracking method that allows addressing whole population activity changes by following individual somata in 3D and over time. Importantly, this method will also make automated 3D detection and activity tracking possible for many additional samples. Our approach offers the potential to acquire a precise count of responsive neurons, as well as to capture the spatial organization of responsive and non-responsive neurons. The application of this approach should also open the door for dedicated rich euclidean graph-based spatial analyses of sensory processing as well as memory formation.

## Methods

### Fly conditioning

For the training protocols, we used the odors 3-octanol (OCT) and 4-methylcyclohexanol (MCH) as the conditioned stimuli. The two odors, which have been widely used for conditioning experiments in *Drosophila*, can both be associated with an appetitive or aversive response.

During training, groups of 50–100 flies were initially exposed for 60 seconds to the first odor (odor A: either 0.36 mM OCT or 0.325 mM MCH diluted in paraffin oil), during which time they received 12 consecutive electric shocks (ES) corresponding to 1.5-second pulses of DC. After a 45-second rest period, flies were exposed for 60 seconds to the second odor (odor B), which was not paired with ES. This training cycle was repeated 5 times, with a 15-min resting interval. Flies were then kept in a vial with regular solid food.

### Dissection protocol

Flies were glued on a plastic slide using a biocompatible dental glue (3 M ESPE Protemp) and pierced in the center, without any prior anesthesia. An alignment wire was used to maintain the Drosophila head in a correct position. The orientation of the head was adapted to the area of interest to be imaged so as to minimize the thickness of tissue the light must travel through.

Next, Drosophila heads were opened using very fine scalpels to remove a rectangular cuticle region (300 µm × 400 µm) covering the brain. The underlying fat tissue was pushed to the corners of this window, and the tracheae were cut and pushed aside to obtain a clear view of the brain. In general, all actions must be performed extremely carefully so as not to damage the glial cells that surround the brain, as well as the MB itself. All microsurgery was performed in the presence of a physiological fluid (Ringer’s solution) to preserve the brain. The composition of this aqueous solution is as follows: 130 mM NaCl, 5 mM KCl, 2 mM MgCl_2_, 2 mM CaCl_2_, 36 mM C_12_H_22_O_11_ (sucrose), and 5 mM HEPES-NaOH (Sigma-Aldrich). The pH of the solution is 7.3^18^.

### Odor delivery system

Two pumps were positioned upstream of the odor delivery system, one of which feeds a pipe circuit controlled by a series of solenoid valves. These valves made it possible to generate different stimulation configurations. The pipes were either immersed in bottles containing neutral paraffin oil for “air defect” and “air control” configurations, or bottles with added chemical product: 4-methylcyclohexanol (MCH, purity equal to 99%, Fluka 66360, Sigma-Aldrich) or octan-3-ol (OCT, purity greater than 95%, Fluka 74878, Sigma-Aldrich). Since these products are hydrophobic, the solutions were prepared in odorless paraffin oil (International VWR, Sigma-Aldrich); we used 1 mL of product dissolved in 100 mL of paraffin oil. The flow exiting this part of the assembly corresponds to one-third of the total flow delivered to the fly. The other two-thirds of the flow were generated by a second pump. This second pump is connected to a pipe immersed in a bottle filled with neutral paraffin oil, which creates a constant main airflow regardless of the chosen stimulation configuration. The final odor concentration arriving at the Drosophila antennae was 1/500.

In order to prevent the odor from stagnating in the delivery chamber, another pump was used to evacuate the odor and avoid desensitization of the Drosophila olfactory receptors. The solenoid valves were individually controlled by logic signals from a NI-USB (National Instruments) card to define the desired pacing configuration.

### Image acquisition and odor presentation

To generate the flies for imaging, female Drosophila carrying the VT50339-Gal4 driver (3rd chromosome) were crossed to males carrying UAS-GCaMP6f/UAS-NLSmCherry (both 2nd chromosome) in order to express the calcium sensor and the nuclear marker in the mushroom body. In our experiments, a Zeiss Examiner Z1 Axio microscope was used for data acquisition, equipped with an EMCCD (Electron Multiplying Charge Coupled Device, Photometrics Delta Evolve). The light excitation was performed by two diode-pumped lasers that emit at 491 nm and 561 nm (maximum power: 50 mW, Roper Scientific). The sample was scanned using a CSUX1-M1N-E confocal head. This Nipkow disc, consisting of a spiral arrangement of 20,000 50-µm diameter filtering holes each spaced at an interval of 250 µm, rotates at a maximum speed of 5,000 rpm synchronously with a second disc made of the same number of micro lenses (diameter: 250 µm). When the discs rotate, approximately 1,000 laser beams simultaneously scan the sample. A set of interference optical filters (model 59022 ET - EGFP/mCherry, Chroma) were used to define the different spectral paths of the microscope, with each of the filters consisting of two transmission bands. The dichroic plate was used to reflect the excitatory light to the sample and transmit the emitted fluorescence to the camera.

A set of mirrors and filters (Dualview Photometrics DV2) were mounted on the transmission path of the microscope upstream of the camera to allow simultaneous acquisition at the camera of two wavelengths (here, mCherryRFP as a nuclei marker and GCamPEGFP to monitor neuronal activity). Two water immersion microscope objectives are available on this device: Zeiss 40x ON 1.0 Vis-IR W apochromat 421462–9900 (working distance: 2.5 mm) and Zeiss 63x ON 1.0 Vis-IR W apochromat 421480–9900 (working distance: 2.1 mm). Although the initial tests were made with the 63x objective, all of the acquired data used the 40x. Finally, the entire system was controlled by the VisiView 2.1.3 software (Visitron Systems GmbH).

Sequences of 3D stacks were saved in a 5-dimensional*.tiff* file, with axis XYZTC and data recorded in a 16-bit format. Each plane was 256 × 512 pixels, as we only used half of the sensor for each channel (the full resolution of the camera is 512 × 512 pixels). The pixel size was 0.16125 µm × 0.16125 µm, and the total size of each 2D image was 41.28 µm × 82.56 µm. The acquisition step size was defined as 1.5 µm, with 45 2D images being sufficient to cover the depth of the whole MB. Therefore, each 3D stack can cover a depth of 67.5 µm. Every 2D image had an exposure time of 20 ms, meaning that each 3D stack required 0.9 seconds to be acquired. Due to the high frequency of acquisition, we did observe an artifact that corrupted the 3D stack acquisition at random times (Supplementary Fig. 14). As this issue arose in a sporadic manner, it was easy to deal with its detection, and the affected time frames were automatically excluded from the analysis and replaced by interpolated data.

In parallel to the image acquisition, flies where exposed to the octanol and air control stimuli as follows: 45 s of airflow - 5 s of air control pulse - 35 s of airflow - 5 s of octanol pulse - 35 s of airflow - 5 s of air control pulse - 35 s of airflow - 5 s of octanol pulse - 35 s of airflow. The whole acquisition time was 225 s (250 frames). To capture odor responses for LTM retrieval, we used only the first air control pulse and octanol pulse for analysis (108 s/ 120 frames). For odor discrimination, the stimuli where presented as follows: 10 s of airflow - 5 s of air control pulse - 35 s of airflow - 5 s of octanol pulse - 35 s of airflow - 5 s of methycyclohexanol pulse - 35 s of airflow - 5 s of octanol pulse - 35 s of airflow - 5 s of methycyclohexanol pulse. The sequences started randomly with Octanol or methylcyclohecxanol. The whole acquisition time was 175 s (180 frames).

### Rigid and non-rigid registration

Registration between two point sets is the process consisting of modifying, as a whole, the positions of the points from one set to make them as close as possible to the points in the other set. An important step of our tracking approach consists of registering all the spot detections of the sequence to a single reference frame in the middle of the sequence. This section explains why and how it is achieved.

Applying spot detection independently on each 3D stack of a sequence produced as many 3D point sets St as time frame: {St, t = 1,.., T}. Because the spot detection step was inaccurate due to the low image resolution and the high density of objects, each somata over time could be alternatively detected as one, several or no 3D point leading data association of regular spot tracking algorithm to fail. Therefore, we took advantage of the fact that in our case, unlike in regular tracking applications, all points belong to the same tissue. Consequently, registering 3D point sets as a whole to cancel movements and group detections over time by somata should help resolving data association even in case of sub- or over-detections. In our case we registered all the stacks of the sequence to a reference frame using rigid (in fact affine), then non rigid registration algorithms.

The purpose of the affine registration was to first cancel large and global shifts due to erratic movements of the fly caused for instance by the pulsatile organ or by the spatial drift. Each 3D point set S_t_ corresponding to each time frame t < T/2 of the first half of the sequence was registered to the point set of the next time frame S_t+1_ by identifying optimal parameters of an affine homogeneous transformation matrix **A**_t_ (including rotation, translation and scaling but not shearing) that minimizes the sum of distances between the two point sets. To this aim, two random subsets of equal size 100 from S_t_ and S_t+1_ were considered and optimally coupled using the Hungarian algorithm^48^ prior minimization. The Hungarian finds the optimal 2D association between two point sets of the same size and the minimization process identifies the affine transform that minimize the sum of squared distance between the 100 couples of point thus defined. The process to align each time frame t to the time frame t + 1 thus reads:

$$\begin{array}{rcl}{{\boldsymbol{S}}}_{{\boldsymbol{t}}}^{{\prime} } & = & {\rm{rand}}({{\boldsymbol{S}}}_{{\boldsymbol{t}}},100)\\ {{\boldsymbol{S}}}_{{\boldsymbol{t}}+1}^{{\prime} } & = & {\rm{rand}}({{\boldsymbol{S}}}_{{\boldsymbol{t}}+1},100)\\ {{\bf{A}}}_{t}^{\ast } & = & \mathop{{\rm{\min }}}\limits_{{{\bf{A}}}_{t}}\sum _{({{\bf{x}}}_{i},\,{{\bf{y}}}_{i})\in {\rm{Hung}}({{\boldsymbol{S}}}_{{\boldsymbol{t}}}^{\text{'}}\,,\,{{\boldsymbol{S}}}_{{\boldsymbol{t}}+1}^{\text{'}})\,}{({{\bf{A}}}_{t}{{\bf{x}}}_{i}-{{\bf{y}}}_{i})}^{2}\end{array}$$

Once all homogeneous transformation matrices obtained {${{\bf{A}}}_{t}^{\ast }$, t = 1 to T/2}, all data points for t < T/2 were transformed to the reference frame t = T/2 chosen to be in the middle of the sequence. To transform any points **x** from an arbitrary time frame t_0_ < T/2 to the reference frame, we chained the homogeneous transformations the following way:

$${T}_{{t}_{0}\to T/2}({\bf{x}})={{\bf{A}}}_{\frac{T}{2\,}-1}^{\ast }{{\bf{A}}}_{\frac{T}{2}-2}^{\ast }\ldots {{\bf{A}}}_{{t}_{0}+1}^{\ast }{{\bf{A}}}_{{t}_{0}}^{\ast }{\bf{x}}$$

The same principle was applied in the reverse way for all time frames t > T/2. At the end of this process, all points from all time frames were roughly aligned to the frame T/2, correcting for the coarse movements of the brain.

This first step could correct for large movements but was not able to correct for smaller, local deformations caused by the elastic property of the tissue. This finer grain correction was performed through an additional non rigid registration we performed using a straightforward application of the Coherent Point Drift (CDP) algorithm^27^. CDP considers the two data point sets to register as realizations of close by Gaussian Mixture Models (GMM). The algorithm forces the GMM to move from one set to the other coherently as a multimodal distribution to preserve the topological structure of the point sets. Unlike with the rigid transformation, each 3D point set **S**_t_ was not registered to **S**_t+1_ but directly to the reference 3D point set **S**_T/2_. This is because the affine registration already corrected for the coarse long term movements. On the other hand, note that CDP could not directly correct for large shifts on its own and the prior rigid registration enabled CDP to properly correct for local deformations. Overall, aligning all 3D detections overtime enabled to form clusters which the following step could detect and use to further identify, split and complete trajectories.

### Reconstruction of trajectories and missing detections

After applying DBSCAN clustering on a registered sequence, the median number of detection per time frame for each cluster was computed. The closest integer of this median value was the number of trajectories included contained in the cluster (with an implicit large tolerance for missing or multiple detections at some time frame). Most clusters produced a value of 1 which made them obvious candidates for individual trajectories. When this value was not one but an integer n > 1, it meant that the cluster contained n trajectories. In this case, it was split into n clusters using the k-means algorithm (see Supplementary Fig. 4). Finally, once all trajectories were identified, missing detection at some time frame t was reconstructed by linear interpolation of the two detections available in frame t − 1 and  t+1. Using this principle, missing detections in consecutive frames could also be recovered by linear interpolation of the closest available detections in time. Reversely, more than one detection at the same time step indicated an over detection and only the detection closest to the centroid cluster was kept (Supplementary Fig. 4). Overall, missing or additional detections at any time steps, even in significant proportions, could not corrupt the reconstruction of trajectories as assessed by our quantitative evaluation.

### Quantitative evaluation for spot detection

To evaluate and compare the accuracy of the spot detection methods, a detection is considered a true positive (TP) when it falls within a sphere of 1.5x nucleus radius around each annotated ground truth position. A detection is considered false positive (FP) when it falls outside all spheres. Note that if two detections fall in the same sphere, only one is accounted as TP, the other one is accounted as FP. A sphere that enclose no detection is accounted for as a False Negative (FN). There is no True Negative (TN) as no other object than nuclei are annotated and no tracking software result contains anything else than nuclei trajectories. As measure of accuracy we used the Jaccard index, a similarity index defined as the ratio between the intersection and the union of two sets. In our case the two sets are defined by the detection results and the ground truth and the Jaccard index value is then TP/(FP + TP + FN). When the intersection of the detection results and the ground truth translates in a high amount of FP or FN relative to the TP, the Jaccard index approaches zero and the detection results is considered bad quality. Oppositely, if perfect match between the detection results and the ground truth, FP = FN = 0 and the Jaccard index value is one. The results for this validation can be seen in Supplementary Fig. 2, for both synthetic and manually annotated images.

### Quantitative evaluation for tracking

Tracking was operated by 3 software programs for evaluation (1) ours: memotrack, (2) ICY^49^ and (3) TrackMate^50^. ICY and TrackMate were chosen both because they were available online and because they received good evaluations from a recent spot tracking performance review^51^. After tracking, trajectories that were interrupted (when their duration were shorter than the total sequence) were discarded as the complete sequence was needed to read the GFP signal. Distances between the remaining trajectories and annotated ground truth (manual or synthetic) were computed and a trajectories with an average distance over time from its closest ground truth larger than 3 times the nucleus size was considered wrong (mostly to allow for the imprecision in the axial direction). Correct trajectories defined this way represented the true positives (TP) in Supplementary Figs. 6 and 7. False Negative (FN) were defined as ground truth nuclei that did not match any trajectories. False Positive (FP) were software defined trajectories that did not match any ground truth. Note that this last category is unavailable for manually annotated data as it would necessitate to annotate all nuclei of a 3D sequence over time (about 250,000 data points!), which is virtually impossible for a human being. Finally, note that there was not such a thing as True Negative (TN) as software programs do not generally output trajectories corresponding to spurious objects that we anyway wouldn’t have annotated.

### Manual annotation

Manual annotation of 3D stacks were performed using the ImageJ “Cell counter” plugin^52^. Two separate annotations were performed. One for the validation of the spot detection on static 3D stacks, and another one for the 3D tracking over time. For the spot detection, all nuclei in one 3D stacks were annotated independently by two experts. It consisted in marking the central position in 3D of every nuclei present in the Mushroom body (mCherryRFP signal). The XY position was relatively easier to be assessed with accuracy, while the lower axial (Z) resolution of the 3D stacks makes the process more difficult. For this last reason, annotated ground truth in 3D cannot be considered as a perfect. For tracking, manual annotations are typically time consuming and don’t guarantee a high accuracy^51^. In our case, it was impossible to annotate every individual object, even in one dataset in an exhaustive manner. Instead, 10 random nuclei were annotated by two experts along a sequence of 250 3D stacks for a total of 5000 data points. One nucleus happened to be chosen by both annotators leading to 19 single nuclei annotated in total. In both cases (detection and tracking) the ground truth was exported in a XML file, so that a straightforward comparison could be done similarly against the results provided by ours and others methods (see Supplementary Figs. 5–7).

### Synthetic data

Static and dynamic 3D stack were artificially designed to validate the spot detection and tracking algorithms. For static 3D stack, a real interpolated 3D stack was used to delineate a foreground using an otsu thresholding. A specified number of spheres were drawn into a similar sized volume and evenly spaced using the k-mean algorithm. These spheres were then convolved with a PSF measured from isolated spots in the original 3D stack to render a fluorescent microscopy 3D stack. Anisotropy was obtained by subsampling the obtained 3D stack in the axial direction. Gaussian noise was finally added and the known coordinates of the spheres were saved as ground truth in a separated file. To generate sequences of 3D stacks to validate the tracking, we used the same approach than with static 3D stacks except that seed points used to generate the 3D stack at time t were the positions obtained at time t − 1. In this way the evolution of the outer bounds surrounding the foreground (that is the shape of the MB) would govern the movements of all somata in a smooth way resembling the natural movements and deformations of the MB during acquisition (see Supplementary Fig. 5 and Supplementary Video 3). The known coordinates of all the spheres overtime were saved as ground truth in a separated file. A straightforward evaluation of our methods and two other methods could then be performed by comparison of all results with this ground truth (see Supplementary Figs. 6 and 7).

### Signal normalization

Raw single neuron signal was normalized prior to selecting responsive neuron in order to align the background signal for all flies of a batch. Raw signal was smoothed (using a Butterworth frequency filter at 20% of the Nyquist frequency) and normalized the standard way with (F-F0)/F0 where F is the raw signal and F0 is the value of a moving average of radius 10 around the point being normalized (windows of 20 time frames). Neurons with normalized signal containing a peak in an OCT stimulation window with a value higher than 0.1 were considered responsive neurons.

### Batch alignment

All samples in this study were acquired in three different batches separated by periods of time, modifications and moving of the spinning disk microscope. Therefore we performed a correction to minimize batch to batch variations also known as batch effect. Correcting for batch effect after acquisition by reference alignment is common in high throughput experiments as microarray gene expression^53^ or high content screening^54^. Each of the three batches contained different conditions. However, batches could be adjusted two by two using alignment of common references. The first batch contained naïve, spaced paired (OCT-OCT), spaced unpaired (OCT-OCT), spaced paired (MCH-OCT) and spaced unpaired (MCH-OCT) flies. The second batch performed after the manuscript review contained only the massed control, for both unpaired and paired flies. The third batch operated two months later contained spaced paired (OCT-OCT), spaced unpaired (OCT-OCT), massed control and the Crammer knockdown control flies. Alignment of batch 2 and 3 was possible using the massed control group as common reference. Alignment of these two batches to batch 1 was possible using the spaced unpaired group (OCT-OCT) as common reference. As the scale of intensities of the peak of responding neurons for the same reference group had a tendency to vary per batch, the correction itself consisted in simply aligning the 99 percentiles of those distributions and applying the correcting ratio similarly to all neuron signal of the remaining condition of the batch. We performed the alignment using the 99 percentiles and not the max of the distribution to avoid outliers.

### Statistical tests and box plots

When the distribution of the statistics to be tested could be considered approximately Gaussian (as the mean intensity) we performed t-tests except when the data points were paired, then we performed a Wilcoxon signed-rank test. When distribution could not be considered approximately Gaussian (as cell count), we used the Mann-Whitney U test when comparing two conditions and the Kruskal-Wallis H tests when comparing more than two conditions together. All tests were two-sided. N are mentioned either on the plot or the figure captions. For all box plots, the box extends from the lower to upper quartile values of the data, with a red line at the median. The upper whisker extends to last datum less than Q3 + 1.5*IQR). Similarly, the lower whisker will extend to the first datum greater than Q1–1.5*IQR, where IQR is the interquartile range (Q3–Q1).

## Supplementary information

Supplementary material.

Supplementary video 1.

Supplementary video 2.

Supplementary video 3.

## Data Availability

The annotated and synthetic datasets used to validate the method can be found here: https://dataverse.harvard.edu/dataset.xhtml?persistentId=doi:10.7910/DVN/MEENDT.

## Code availability

The code of this method, which enables reproducing these results and the tracking evaluation on annotated and synthetic data, is available from the following Github repository: https://github.com/biocompibens/memotrack.
