# Supplementary material for: In vivo large-scale analysis of Drosophila neuronal calcium traces by automated tracking of single somata
Source: Sci Rep. 2020 Apr 28;10:7153. doi: 10.1038/s41598-020-64060-x (PMC7188892; doi:10.1038/s41598-020-64060-x)
Supplement: Supplementary file 1 — Supplementary material. [file 41598_2020_64060_MOESM1_ESM.pdf]

Supplementary material

In vivo large-scale analysis of Drosophila neuronal calcium traces by automated tracking of single somata

Felipe Delestro, Lisa Scheunemann, Mélanie Pedrazzani, Paul Tchenio, Thomas Preat and Auguste Genovesio

Supplementary Figures

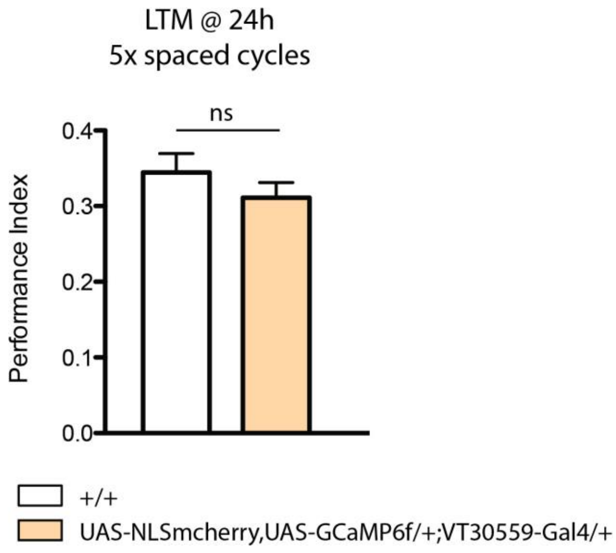

Supplementary Figure 1. Long-term memory scores for wild-type and transgenic flies used during conditioning and image acquisition. A value of zero indicates a random decision of the fly between the paired and control odors. Bars represent standard error. Transgenic flies displayed normal LTM performances.

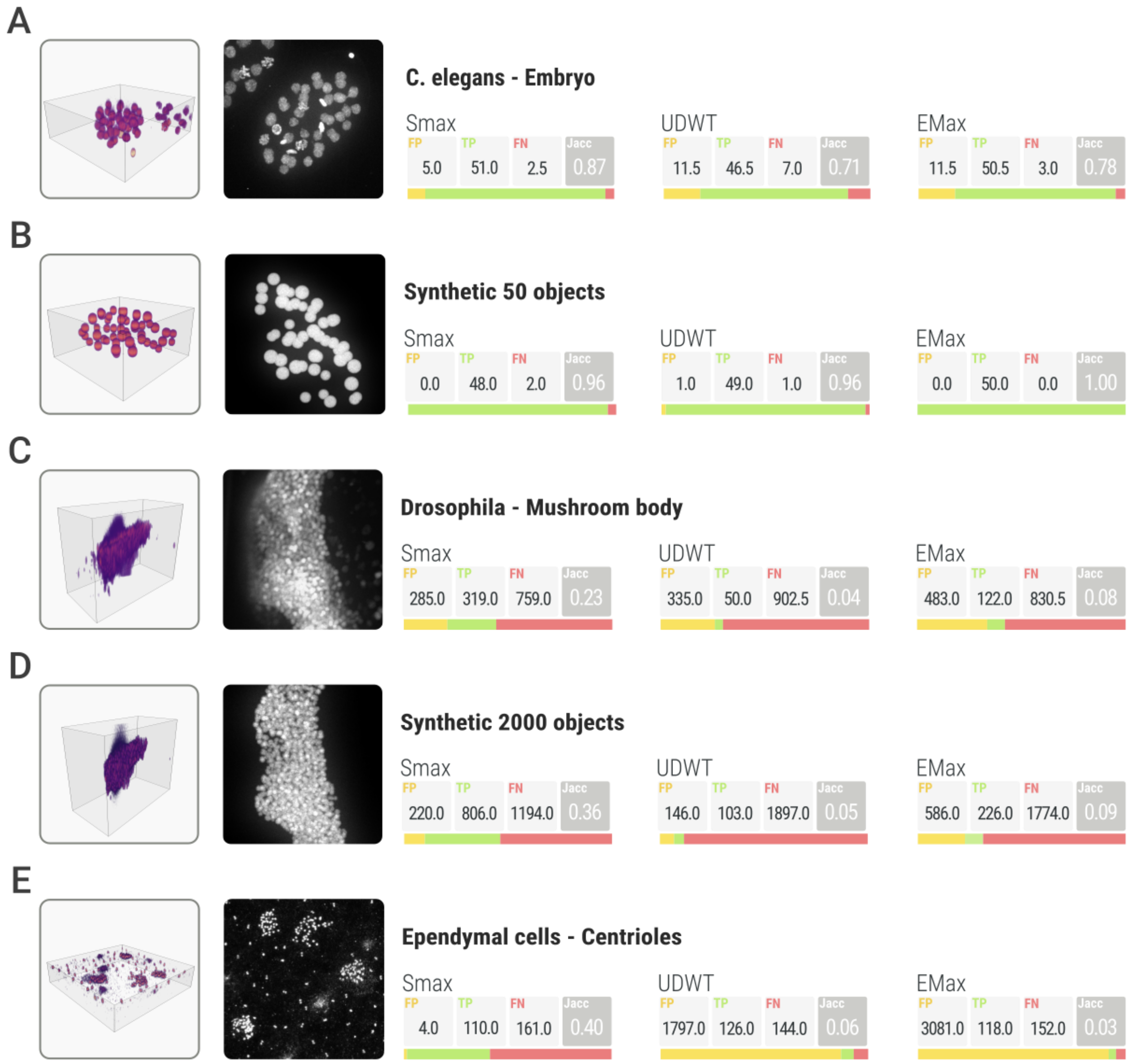

Supplementary Figure 2. Quantitative comparison of our spot detection method alone (without tracking) against two other state of the art spot detection methods on a range of examples. Our approach, called *Smax* here, is compared to the UDWT [38] and eMax [39] using 5 datasets to demonstrate that it is particularly efficient at adressing the case where nuclei are densely packed. The two other methods were promoted in a recent review on spot detection [37]. They are considered efficient and only require a few parameters. Importantly, they are widely used. **(A)** A 3D stack of *C. elegans* with manual annotation of distinguishable nuclei. **(B)** A synthetic reproduction where ground truth is known. **(C)** A 3D stack of a Drosophila mushroom body with a large quantity of manually annotated packed nuclei. **(D)** A synthetic reproduction where ground truth is known. **(E)** A set of manually annotated centrioles from ependymal cells. A volumetric reconstruction and an axial maximum intensity projection are shown for each 3D stack. In each row, the results box indicates (from left to right) the values of false positives (FP), true positives (TP), false negatives (FN; colored bars indicating the proportion for each case) and the Jaccard index (Jacc). TP+FN is either the ground truth count for synthetic data or is assessed manually for real 3D stack. FP+TP corresponds to the detected objects count. A detection is considered positive when it falls in a sphere the size of a nucleus, around any of the original nuclei positions. The sole parameter of Smax was set to the average nucleus diameter which can be easily estimated from the data. Parameters for the two other methods were chosen to be the best possible: a large range of parameters were tried automatically in order to choose the combination that produces the detections that best match the ground truth (best Jaccard index). Importantly, the ground truth must then be known

to systematically scan for parameters and choose the best set, so in principle choosing the parameters of the two others method this way disadvantaged our own approach that does not require any parameter settings. We chose to scan parameter ranges such that the difference of results which looks large could not be blamed on missettings the other methods' parameters: we objectively selected the best ones for the two other approaches.

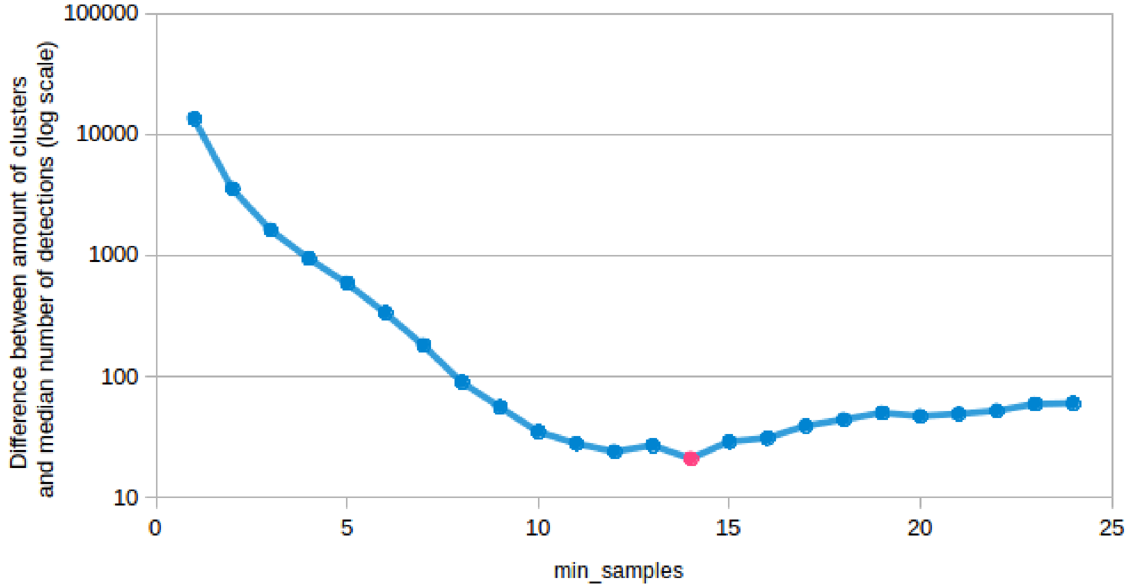

Supplementary Figure 3. Parameters estimation for the density-based algorithm DBSCAN. DBSCAN is a clustering algorithm that computes local density to identify clusters. This method is also able to handle noise by design, meaning that not every point in the dataset will necessarily be part of a given cluster in opposition to widely used algorithms such as k-means or Gaussian mixture models. These characteristics make this approach highly relevant to our problematic. The algorithm consists in an iterative process that uses two parameters to define density: ε (a distance measurement) and *min_samples* (a minimum amount of points). From our data, it was possible to estimate the best parameters for clustering. ε is directly related to the size of the nuclei, so we set it to the FWHM obtained from the average nucleus size at the detection step. On the other hand, the minimum number of points could not be directly estimated as the ε value, mainly because it depends on several uncontrolled factors such as the movement of the brain and the proportion of missing detections in a given time frames. We solved this problem through an iterative process. First, we assumed that the final number of detected clusters should be close to the median number of detections over time. This is a reasonable hypothesis since the number of neurons does not change through time. Then, the DBSCAN algorithm is iteratively applied for increasing values of *min_samples*. The min_samples value that best match the median number of detections over time is chosen as parameter for DBSCAN (indicated by the pink dot in the figure example).

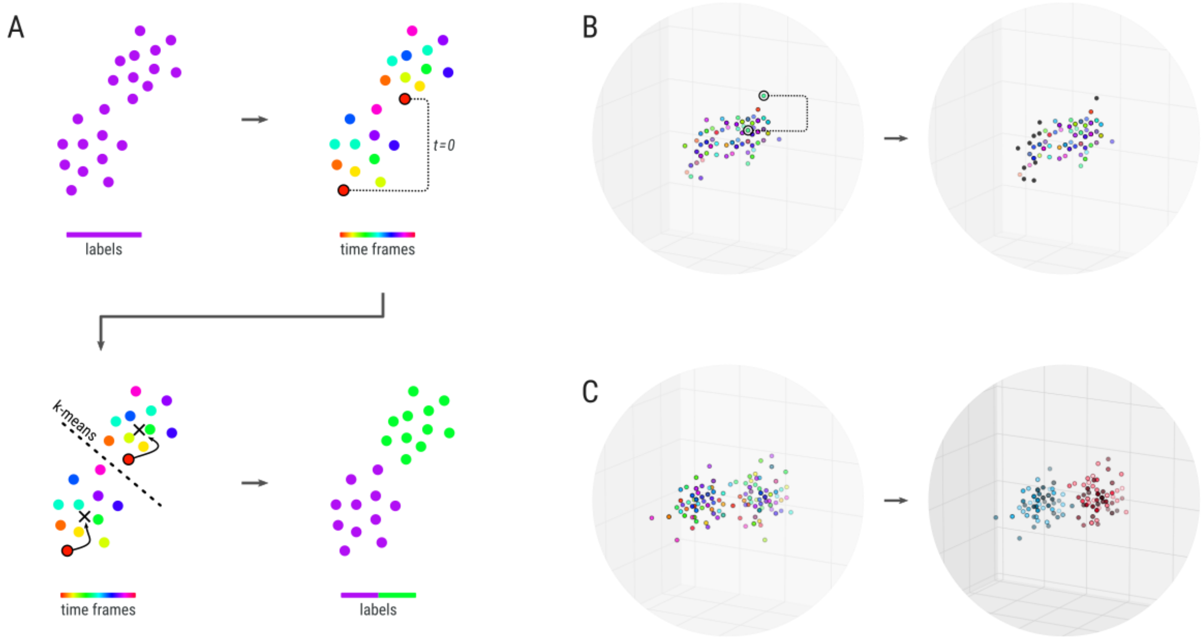

Supplementary Figure 4. Time information improves the quality of clusters. (**A**) A schematic representation of a case where the median number of time frames in a cluster is 2, meaning it contains 2 trajectories. The cluster is then automatically split. (**B**) Real cluster where the median number of time frames is 1, meaning it contains only one trajectory. Then duplicates (the points connected by a dash line) are marked as noise (black dots on the right image) and the point closest to the centroid cluster is kept while the other is discarded from the trajectory. (**C**) Real cluster where the median number of time frames is 2, with the resulting split cluster (in blue and red).

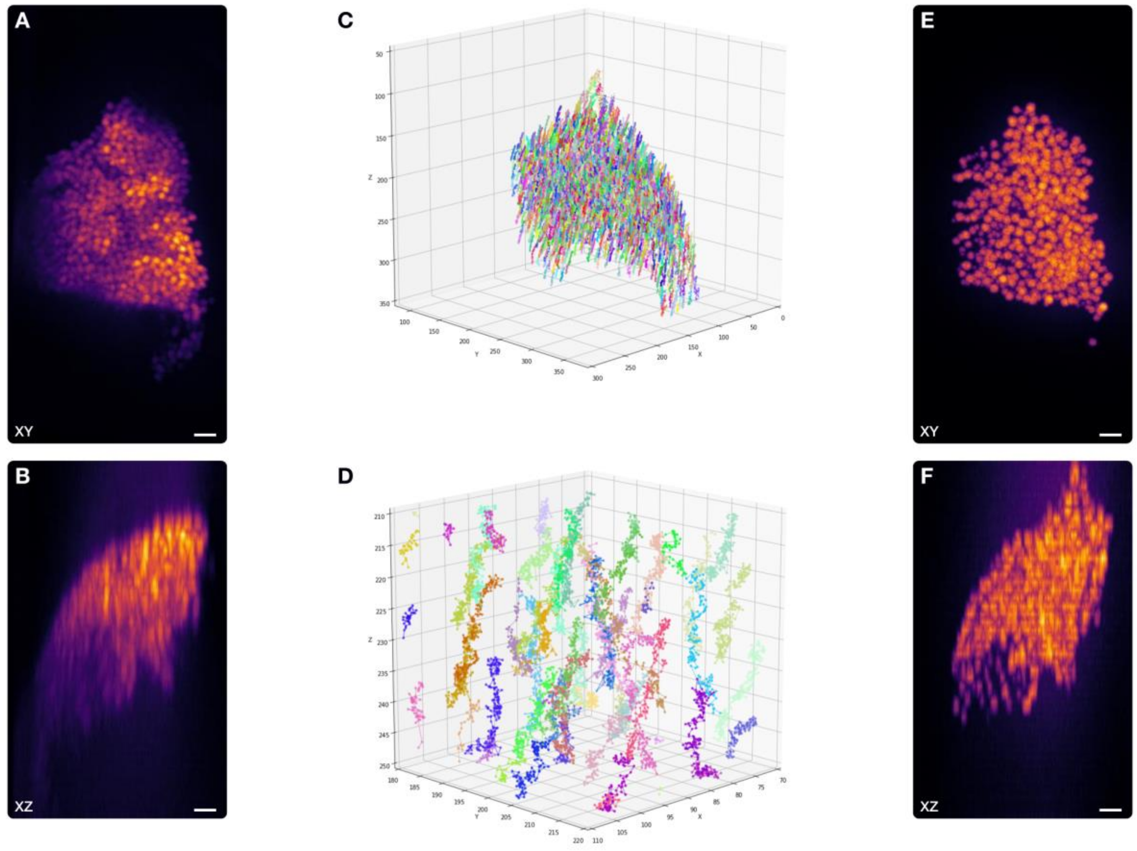

Supplementary Figure 5. Synthetic 3D+time stacks of densely packed nuclei to evaluate the tracking performance against other methods. (**A**) Maximum intensity projection of a real 3D stack that provides a foreground region to generate synthetic somata, along the Z axis. (**B**) Maximum intensity projection of the same 3D stack along the Y axis. (**C**) 2000 generated trajectories in successive volumes over time (see online methods). (**D**) Detail of a central region of the generated trajectories. (**E**) Maximum intensity projection of a synthetic 3D+time sequence generated by convolution and noise addition from the generated trajectories, along the Z axis. (**F**) Maximum intensity projection of the same synthetic 3D stack along the Y axis. Scale bars are 10µm. Although synthetic 3D + time sequences are close to the real 3D + time sequences, they are of course not similar, but a great advantage over manual annotation is that the ground truth is known and all (2000) nuclei are then annotated. Therefore they enable to provide satisfactory quantitative comparisons between algorithms for detection and tracking. Importantly, they enable to quantify False Positive (Supplementary Figure 6).

MANUAL ANNOTATION

A Only complete tracks

Result	TP	FP	FN	Ground	Jacc	Method
482	18	n/a	1	19	0.95	Memotrack
96	8	n/a	11	19	0.42	ICY
0	0	n/a	19	19	0.00	TrackMate

B With incomplete tracks

Result	TP	FP	FN	Ground	Jacc	Method
482	18	n/a	1	19	0.95	Memotrack
192	14	n/a	5	19	0.74	ICY
52	4	n/a	15	19	0.21	TrackMate

SYNTHETIC IMAGES

C Only complete tracks

Method	Jacc	Result	TP	FP	FN	Ground
Memotrack	0.53	1064	1060	4	936	2000
ICY	0.02	38	37	1	1962	2000
TrackMate	0.00	0	0	0	2000	2000

D With incomplete tracks

Method	Jacc	Result	TP	FP	FN	Ground
Memotrack	0.53	1064	1060	4	936	2000
ICY	0.13	306	253	53	1694	2000
TrackMate	0.06	304	119	185	1696	2000

Supplementary Figure 6. Validation of our tracking approach Memotrack against two other methods, ICY and TrackMate, using manual annotated and synthetic 3D+time sequences. **A.** Results obtained using manual annotations and considering only complete trajectories along the whole sequence. That is, if the duration of a trajectory provided by a software program was less than the length of the sequence, it was discarded. This is because the signal needs to be captured along the whole sequence, not during a subpart of it. TP is True Positive, FP is False Positive, FN is False Negative, Result is the output of a software and Ground is the ground truth. Note that False Positive are not available for manual annotation because it was impossible to annotate exhaustively all trajectories of a 3D+time sequence. Memotrack, our method, outperforms other methods with 18 out of the 19 annotated trajectory correctly retrieved. **B.** Results obtained using manual annotations and considering trajectories with length at least as long as half of the whole sequence. This relax in stringency increases the number of successfully tracked nuclei by other software. Those results would not be acceptable or even useful as such to monitor the signal all along the sequence but they enable to understand partly the weakness of the other approaches. Other approaches cannot track nuclei over a long time period without failing because of the low accuracy of spot detection. Our approach, that rely on the non rigid registration of the whole sequence is very robust to detection errors and actually tracks all nuclei that were successfully detected enough time to form a cluster. For the same reason, the length threshold cannot improve the result obtained by our approach as all trajectories retrieved is the length of the full sequence. **C.** Results obtained using synthetic annotations and considering only complete trajectories along the whole sequence. Memotrack, our method, outperforms other methods. **D.** Results obtained using synthetic annotations and considering trajectories with length at least as long as half of the whole sequence. Interestingly, while unusable, we see here that this relax in stringency increases the number of tracked nuclei by other methods but also increases the number of false positive, indicating that even small trajectories provided by those software program are not necessarily correct.

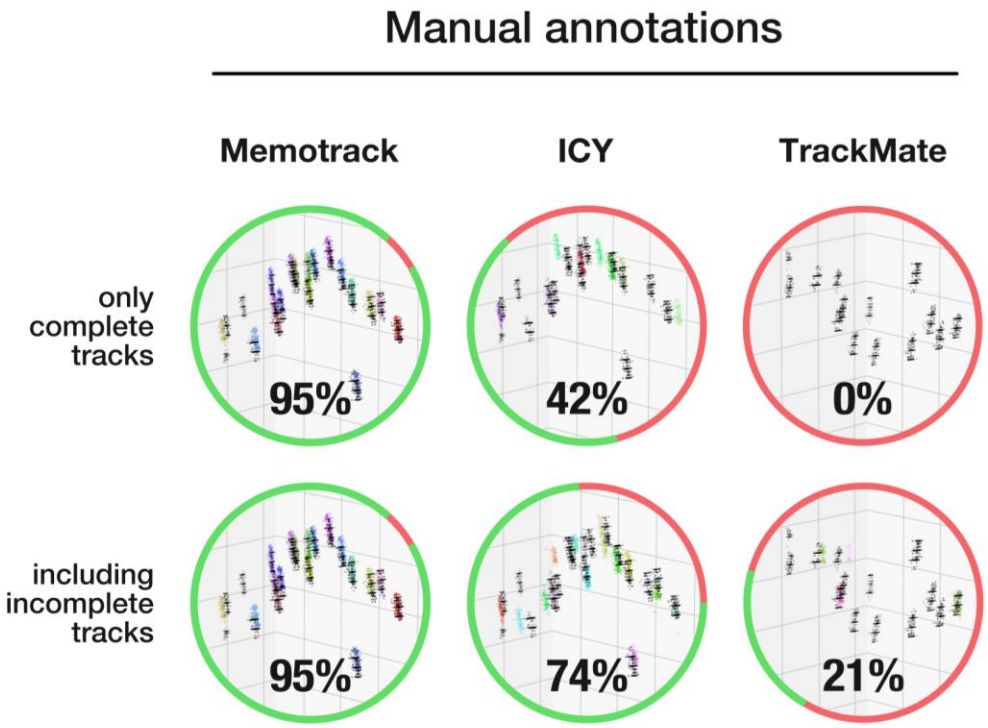

Supplementary Figure 7. Visualization of manually annotated nuclei trajectories (in black, see online methods) and their corresponding trajectories obtained by the tracking software (in color). Top row: only complete trajectories that last the whole sequence were kept, it is the case we were interested in to monitor the single cell signal all along the sequence. We can see that, beyond the fact our method tracks correctly most manually annotated nuclei, the closest trajectories provided by ICY may in fact match other nuclei and be False Positives, an hypothesis that cannot be validated or unvalidated because it was impossible to manually annotate all trajectories in the sequence of 3D stacks. Bottom row: result when we allowed the length of the trajectories to be shorter but at least as long as half of the sequence. Again, those trajectories could not be used for the analysis as they are too short but underline the main limitation of other approaches: other approaches cannot track object stably over a long period of time due to the unreliability of the spot detection step.

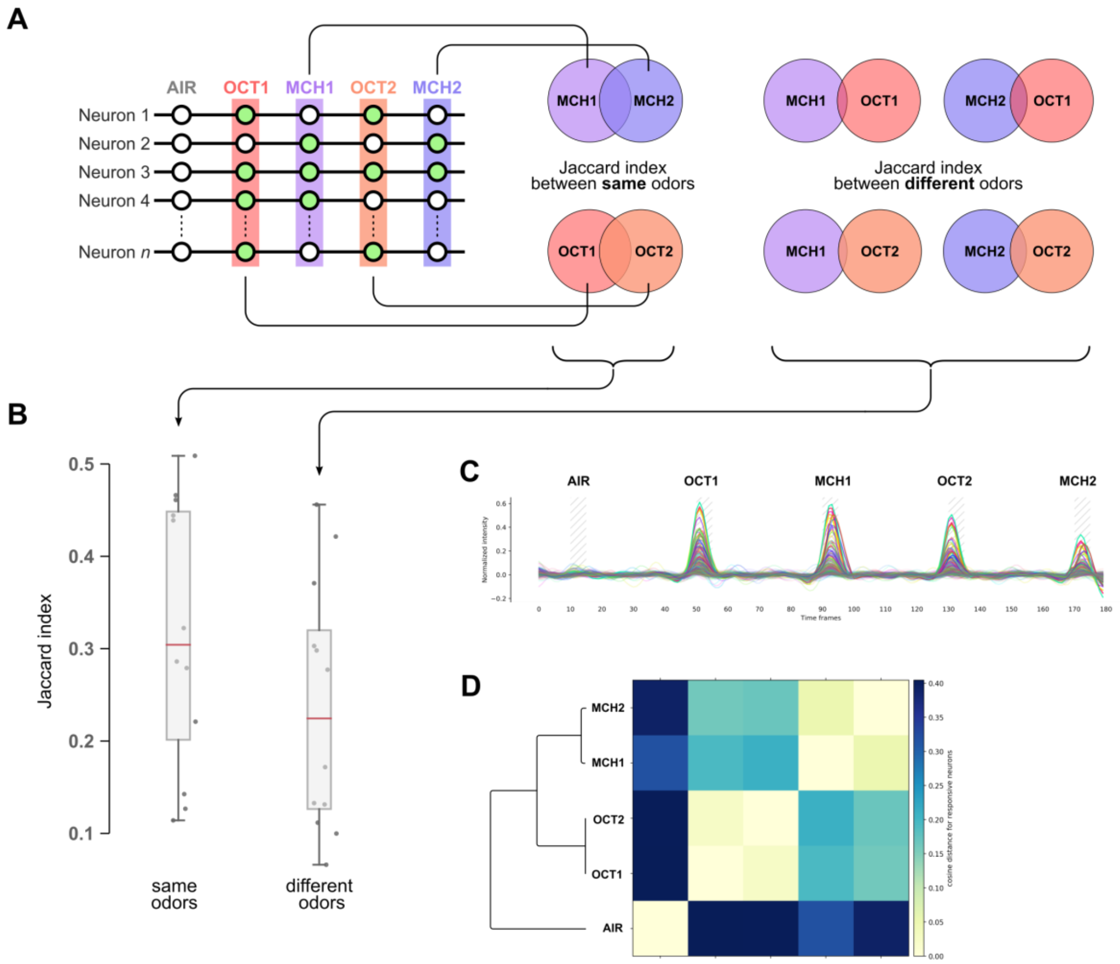

Supplementary Figure 8. Comparison of response between different odors. (**A**) Schematic representation of two mean Jaccard index were collected from each fly to produce: 1) a similarity measure between two presentations of the same odor and 2) a similarity measure between two presentations of different odors. Neurons are represented by rows, and odor stimulation by columns labeled AIR, OCT or MCH and a digit 1 or 2 numbering the presentations. A green disk indicates that the neuron was detected as firing while a white disk indicates that it wasn't. For each fly, the Jaccard indices between two presentations of the same odor (MCH1 vs MCH2 and OCT1 vs OCT2) and between presentation of two different odors (MCH1 vs OCT1, MCH2 vs OCT1 etc) were computed and separately averaged, producing 2 Jaccard indices per fly. The higher the Jaccard index value, the more similar the neuronal response between two presentations on the same fly. (**B**) Distribution of the two Jaccard indices on all flies that passed the quality filter (n=12 flies, paired t-test with p-value 0.0043707). This comparison shows that while some neurons respond to both odors, some neurons are odor specific and pull the Jaccard index to be significantly higher for two presentations of the same odor. (**C**) One example fly from the dataset for illustration, on which each trace corresponds to the signal of one individual neuron, and stimulus regions are marked with hatched bars. (**D**) Clustering of the signals from responsive neurons of the example fly in C using the cosine distance. This clearly shows that neuronal signal clusters per odor presentations and that single neurons responding exclusively to a given odor can be identified using our approach.

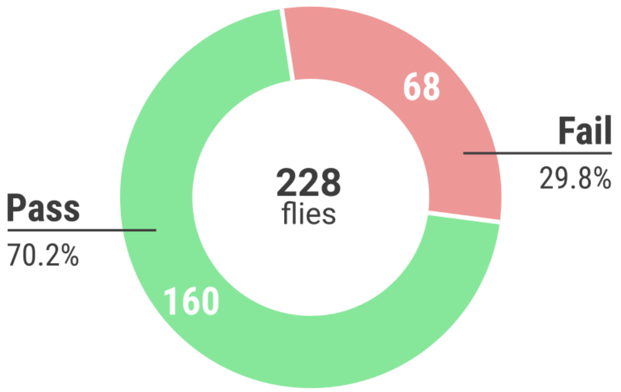

Supplementary Figure 9. Automated fly filter. In some acquisitions, the KCs didn't show any response to the odor, while other acquisitions displayed a continuously erratic response. These are invalid acquisitions that were presumably caused by errors during the fly preparation. To avoid biases of a manual selection from the datasets, we developed an automated procedure to sort the flies. It consisted in comparing the distribution of responsive neurons within the window of octanol stimulation and the initial control window at the beginning of the sequence, using a Mann-Whitney rank test. Flies with a p-value higher than 0.01 were excluded ("Fail"), the remaining flies were used for the analyses ("Pass"). This process guaranteed that the analysis was performed using flies that showed a biological response. Note that for the control experiment with several odor presentations in a row to the same fly, we kept only those flies that passed this filter test for all odor presentations. As this strategy increased drastically the stringency of the filter for these longer sequences, we excluded it from this plot that brings together regular samples with only one odor presentation.

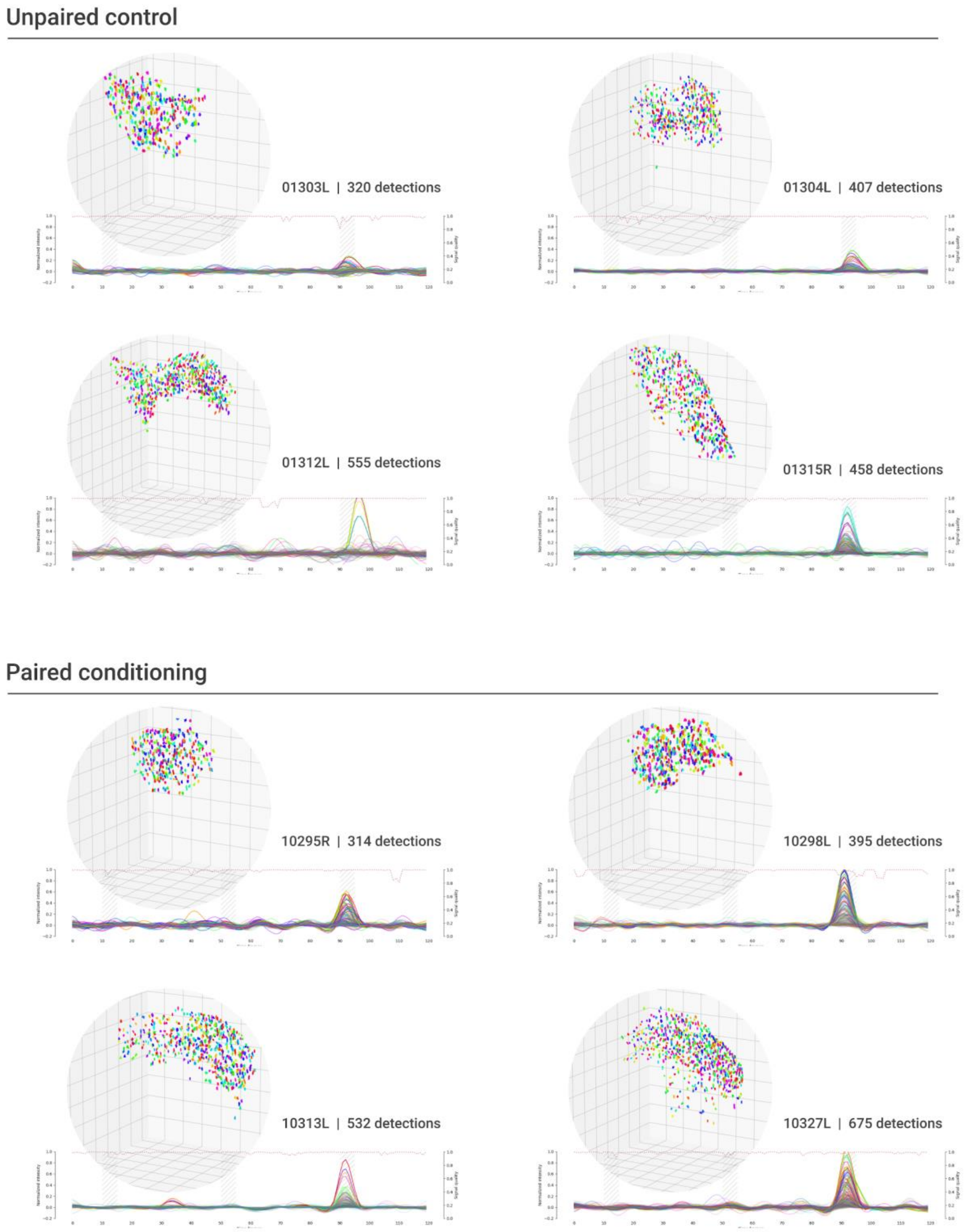

Supplementary Figure 10. Visualization of a few examples of analyzed flies. The top group shows examples from the unpaired control, whereas the bottom group shows flies subjected to paired conditioning. The 3D scatterplot illustrates the tracked detections for each fly (comprising a projection of all time frames to the 3D space); each track is individually colored. The line plot directly under each scatterplot shows the normalized signal measured for each neuron. The three hashed bars indicate the control regions: the first is no stimulus, the second is air and the third is octanol.

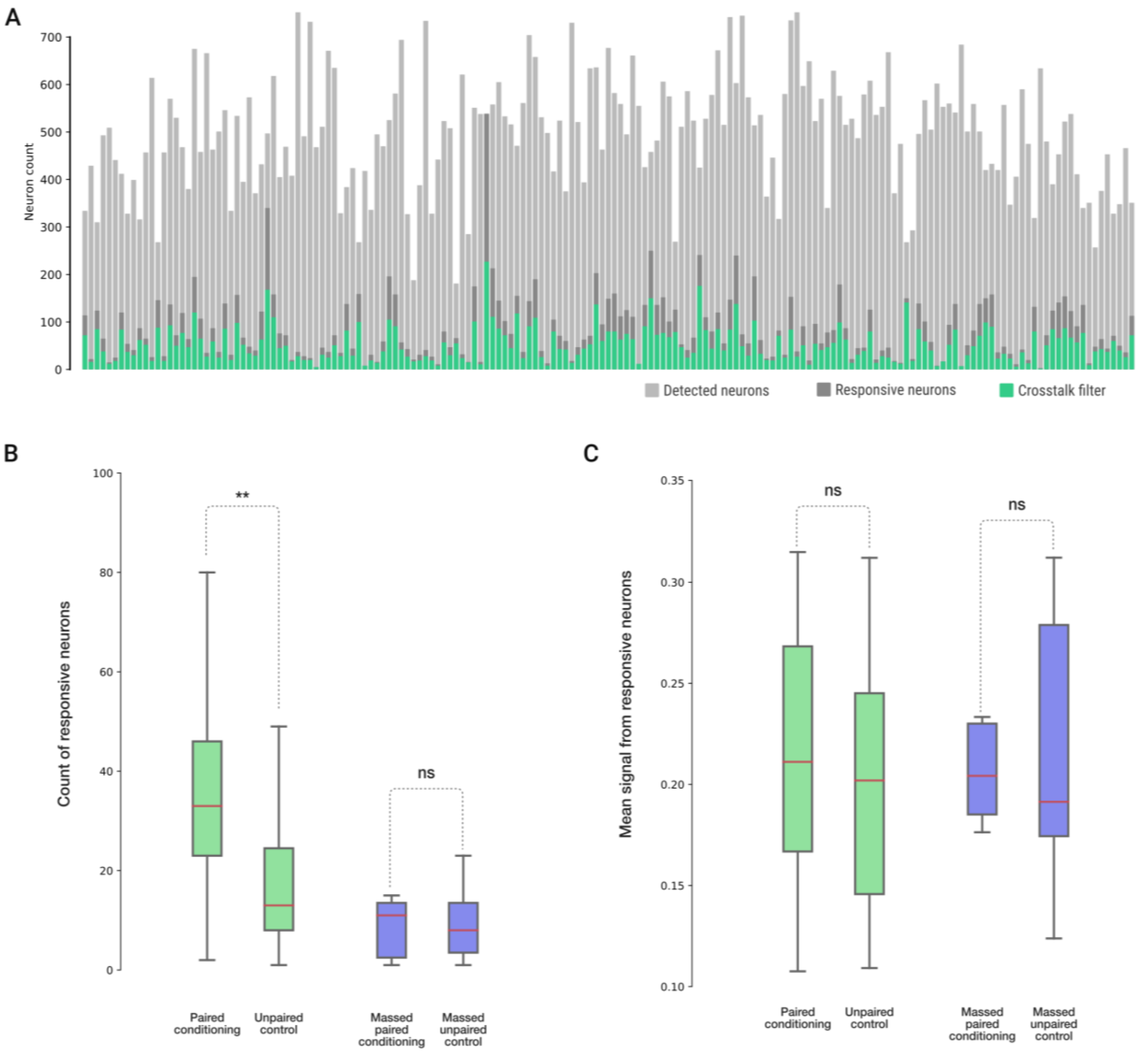

Supplementary Figure 11. In this control, we rule out the possibility for the observed increase in neuron count to be an artefact due to an increase in neighboring neuron intensity by applying a “crosstalk filter”. From each group of closeby responsive neurons (defined as neurons closer than twice the diameter of the soma), only the neuron with the highest signal was kept. Thus the crosstalk filter discarded possibly suspectful neuron from the analysis, artificially ensuring that no neuron counted as responsive could be located close to one another. (**A**) Summary of the dataset, where each column corresponds to one fly. The light gray color shows the number of detected neurons, dark gray is the amount of responsive neurons (17.1% of total detections in average), and green represents only those neurons that passed the so called crosstalk filter, or in other words were kept (63% of the responsive neurons in average). (**B**) The same distributions as in Figure 3, after applying the crosstalk filter (and thus removing with high stringency nuclei that might be erroneously selected as responsive), show that the difference in count between the group trained with OCT and its control is conserved (p-value: 0.0014992), and that the difference in count remains non-significant for the control group with massed training (p-value: 0.881899). A two sided Mann-Whitney test was performed in both cases. (**C**) Furthermore, mean signal intensities from responsive neurons still shows no statistically significant differences, both for the group trained with OCT (p-value: 0.712850) and for the massed control group (p-value: 0.958129). A two sided t-test was performed in both cases. The sample sizes from the 4 groups (from left to right) are 29, 27, 14 and 16.

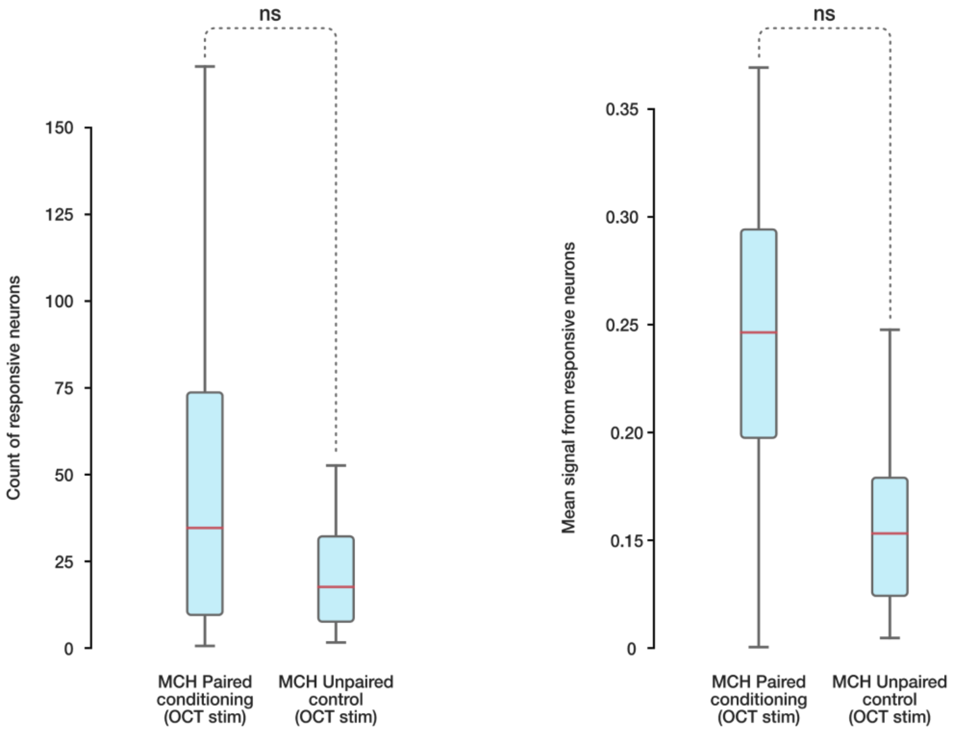

Supplementary Figure 12. Responsive neurons of flies trained with MCH and tested with OCT. Left: There is no significant difference in the count of OCT-responding neurons between the group trained with MCH and its unpaired control (Mann-Whitney test two-sided, p-value: 0.363336). During the conditioning, flies that received MCH also received OCT (albeit without the presentation of shocks). Right: Response intensities do not differ significantly between the MCH-trained paired and unpaired group (Mean signal comparison t-test p-value: 0.067987). The sample sizes is 11 for the paired and 15 for the unpaired group.

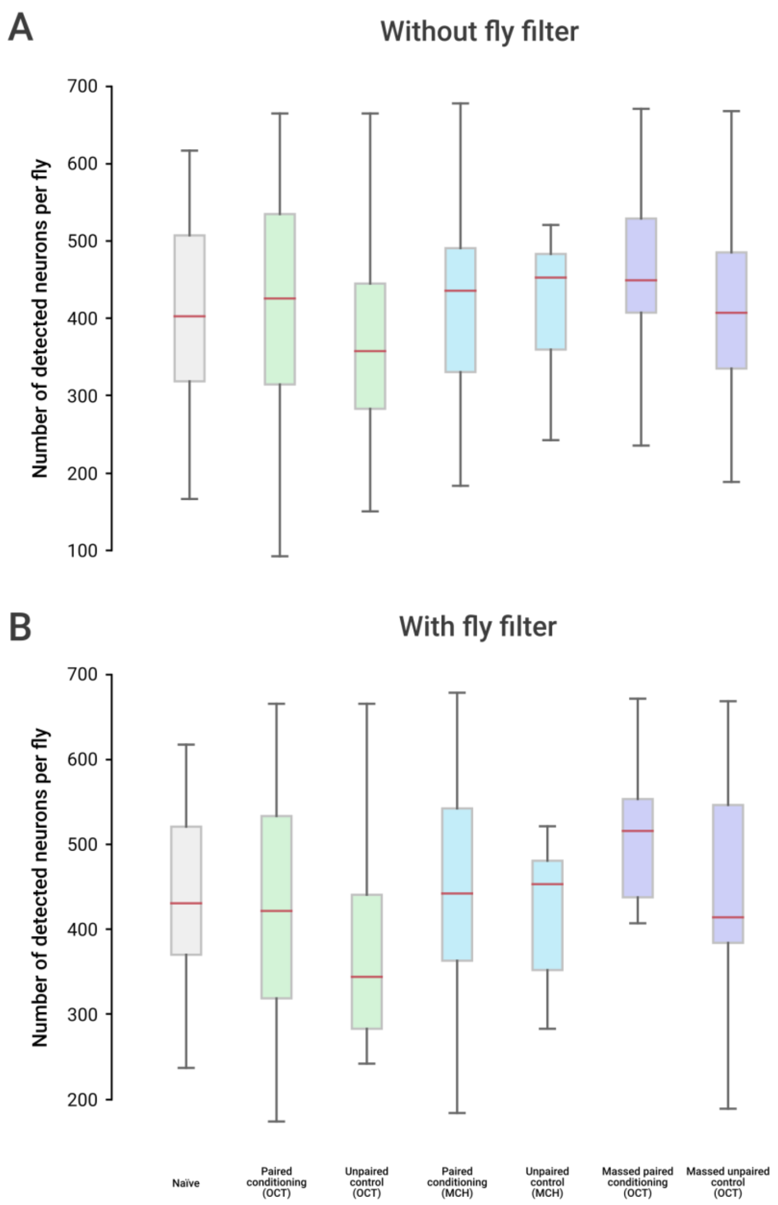

Supplementary Figure 13. The total number of detected neurons is stable before and after applying our quality control filter. (**A**) The total amount of soma detected using the Gal4-driver channel for all tested groups does not show any significant difference between the datasets (Kruskal-Wallis H-test p-value: 0.303595). The sample sizes from the 9 groups (from left to right) are 23, 37, 44, 22, 23, 24 and 22. (**B**) The same distributions as in (A), but restricted to the flies that passed the automated quality control filter (Kruskal-Wallis H-test p-value: 0.1549522). The sample sizes from the 7 groups (from left to right) are 14, 30, 31, 12, 15, 16 and 17.

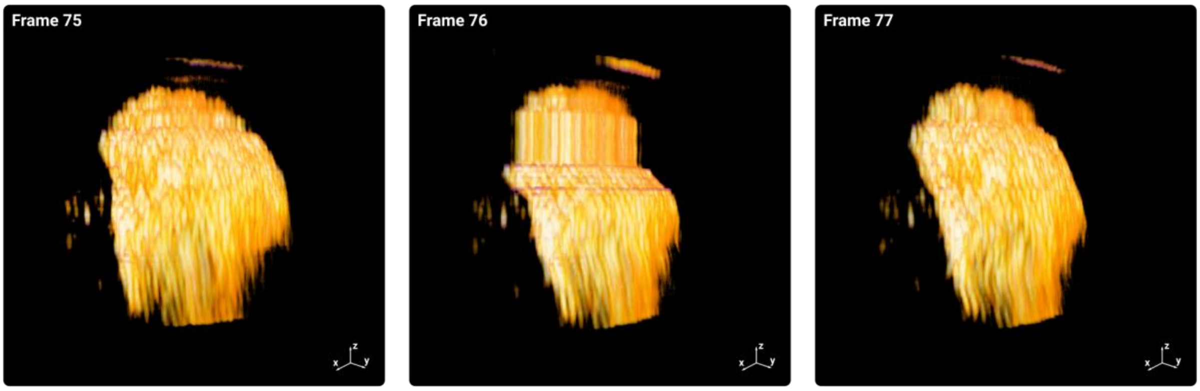

Supplementary Figure 14. Detection of 3D stack artifacts. A 3D volumetric reconstruction of the *mCherry* channel is shown for three consecutive time frames; the artifact of an anchored z position is noticeable in the middle time frame. To automatically detect the artifact, we made the following assumptions: 1) during a normal acquisition, the mushroom body center of mass (based on the set of detected nuclei) should only move slightly. 2) Since a considerable part of the MB was missing when this artifact arose, there should be drastic shift in the center of mass at this time frame. Thus, we measured the derivative of the centroid position through time, normalized to a range between 0 and 1. This quality measurement can then work as an indirect way to identify the time frames in which there was a microscope artifact. A threshold must still be set as the minimum quality level that can be used for the analysis; by visually comparing the quality measurement and the 3D stacks, the value was set at 80%. Time frames with lower values were thus removed, and the missing neuron positions interpolated in time.

Supplementary Videos

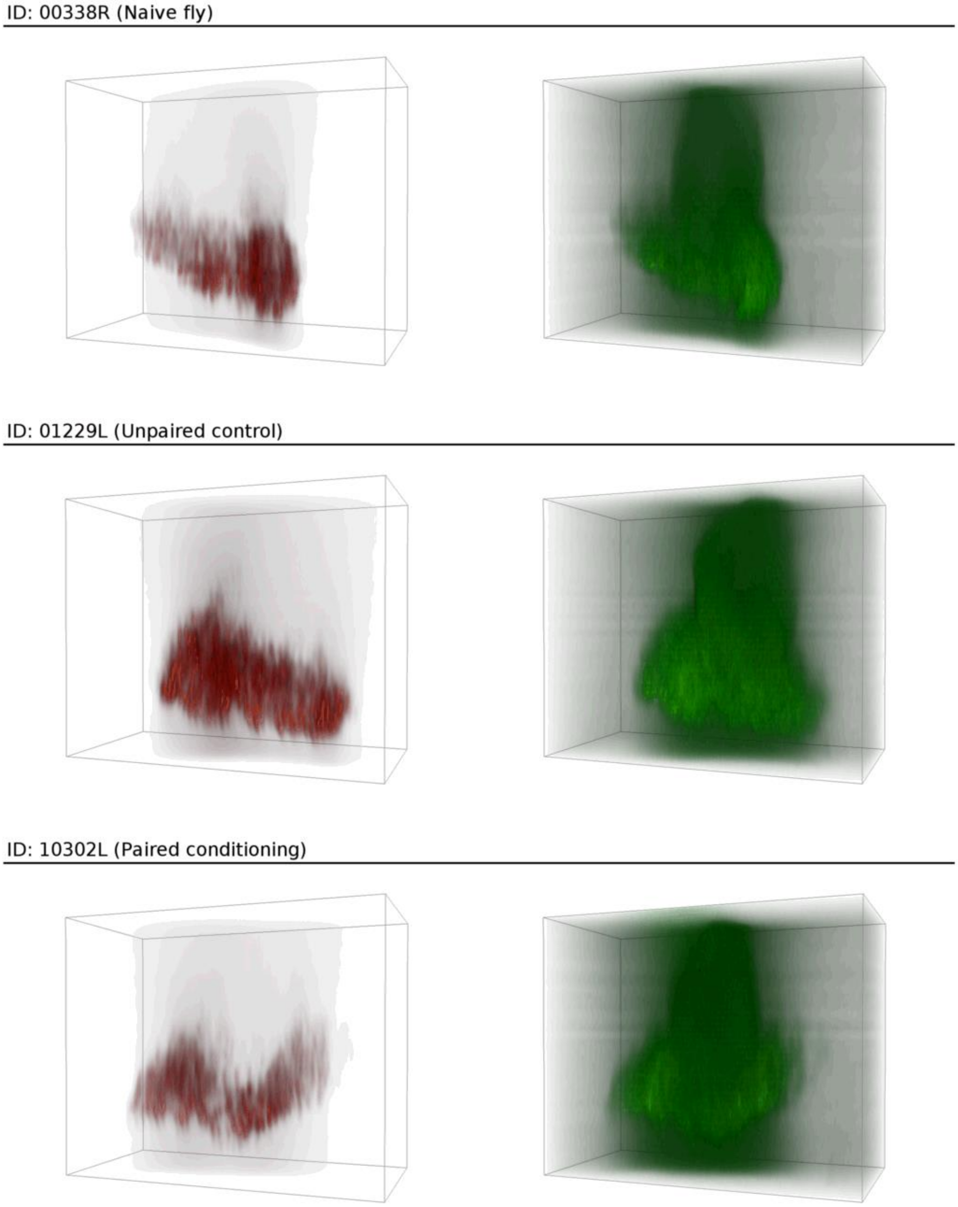

Supplementary Video 1. Volumetric rendering of the raw acquired data. The videos on the left represent the nuclei of the mushroom body neurons, while the videos on the right portray the GCaMP activity of those neurons. The appearance of 'OCT' at the top right of each row corresponds to the time when flies received an octanol stimulation.

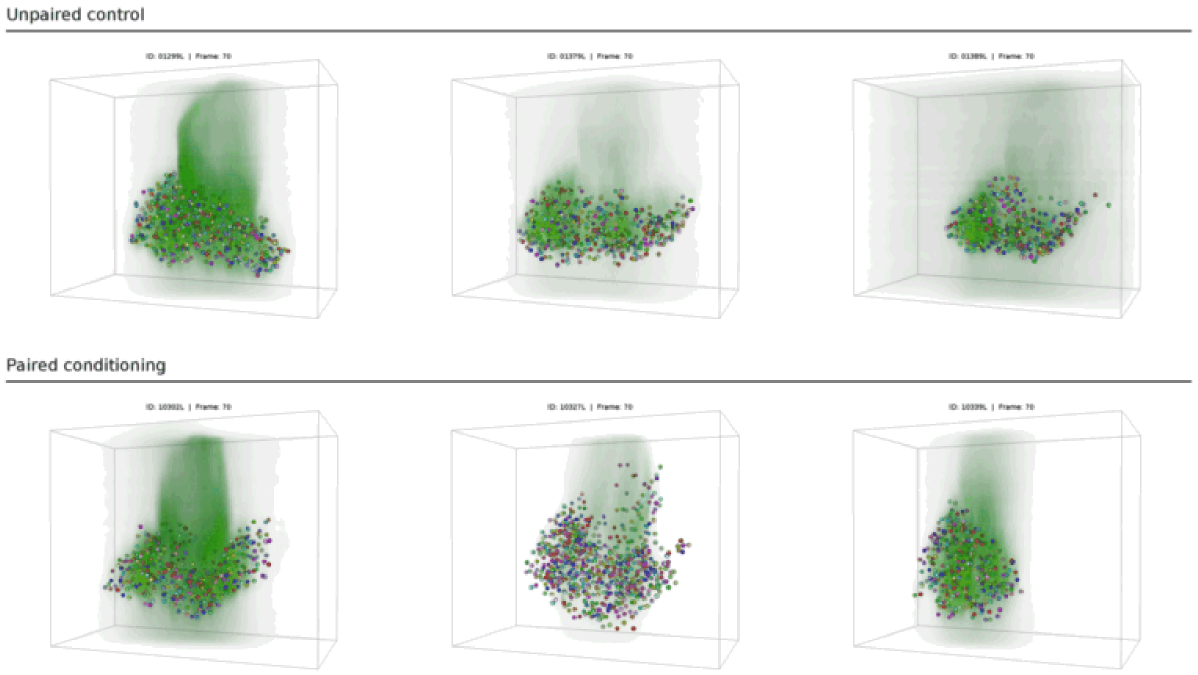

Supplementary Video 2. Volumetric rendering of GCaMP activity together with tracked neurons. The top row shows the signal for three flies from the unpaired control group, while the bottom row shows three flies from the group that received the paired conditioning. Flies received the octanol stimulation starting at frame 90 (corresponding to the appearance of 'OCT' at the top right of each row).

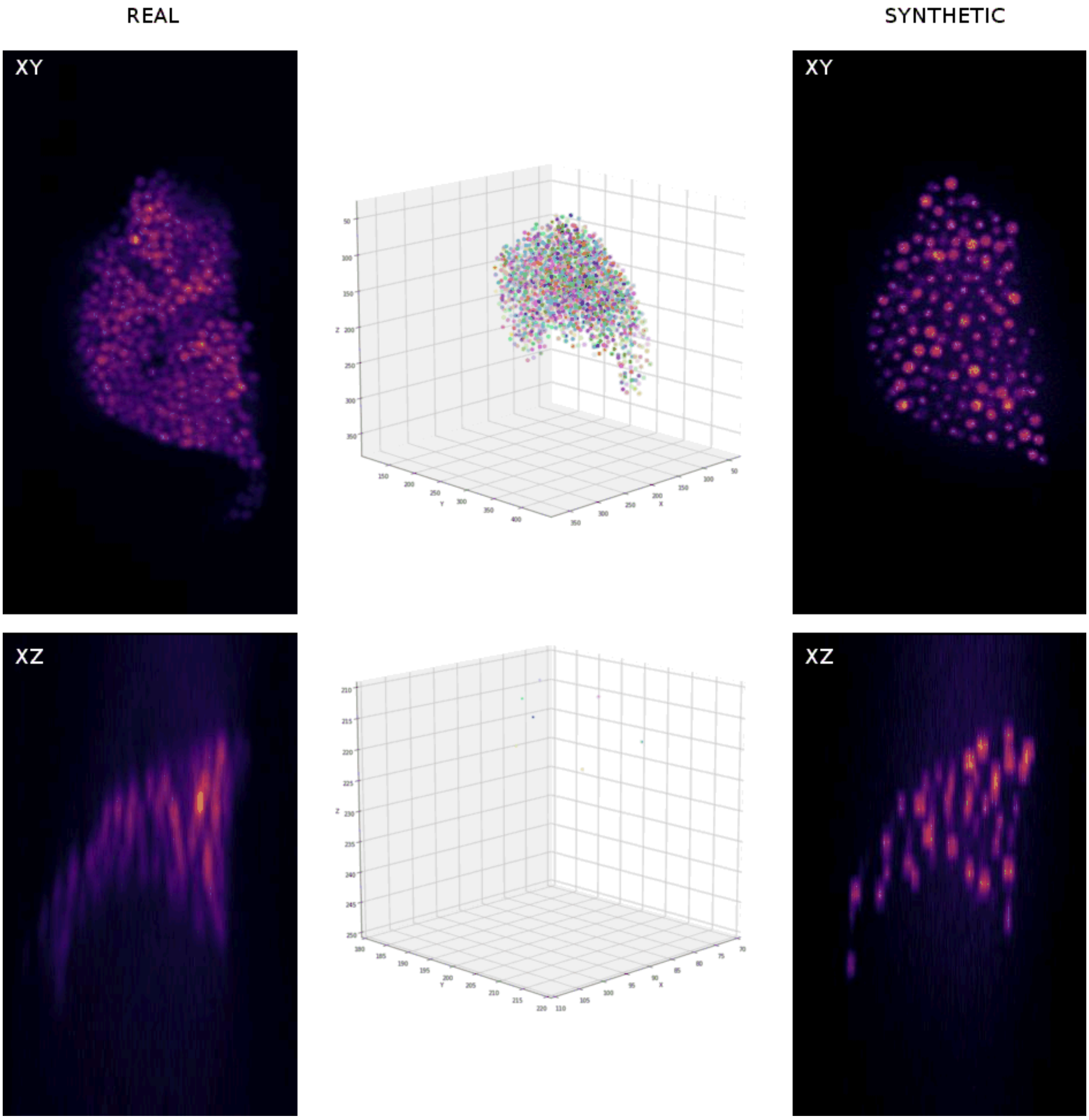

Supplementary Video 3. Generation of synthetic sequence of 3D stacks. Left column shows the middle 2D image (not a maximum intensity projection) from a real 3D stack, in XY (top) and XZ (bottom) views over time. Panels in the middle column shows the generated synthetic coordinates (see online methods) based on the real 3D stack on the left. The top panel shows an overview of all generated 3D positions overtime corresponding to the MB cell bodies on the left. The bottom panel shows a zoomed version inside the synthetic cloud and synthetic trajectories are drawn over time. Right column shows the middle image of the resulting synthetic sequence obtained by convolving the synthetic positions with the PSF of the microscope, adding noise and a similar subsampling to reproduce the anisotropy of the real sequence of 3D stacks, in XY (top) and XZ (bottom) views over time.
